# Application value of whole exome sequencing in screening and identifying novel mutations of hypopharyngeal cancer

**DOI:** 10.1038/s41598-022-27273-w

**Published:** 2023-01-03

**Authors:** Jingwei Yao, Yubo Ding, Xiong Liu, Jialu Huang, Minghui Zhang, Yu Zhang, Yufan Lv, Zhuoyi Xie, Jianhong Zuo

**Affiliations:** 1 Gastroenterology Department, The Affiliated Nanhua Hospital of University of South China, Hengyang, 421001 Hunan People’s Republic of China; 2 Department of Hematology, The First Affiliated Hospital of Xiamen University and Institute of Hematology, School of Medicine, Xiamen University, Xiamen, 361003 People’s Republic of China; 3 Transformation Research Lab, Hunan Province Key Laboratory of Tumor Cellular and Molecular Pathology, Hengyang Medical School, University of South China, Hengyang, 421001 Hunan People’s Republic of China; 4 Clinical Laboratory, The Third Affiliated Hospital of University of South China, Hengyang, 421000 Hunan People’s Republic of China; 5 Department of Otolaryngology-Head and Neck Surgery, Nanfang Hospital, Southern Medical University, Guangzhou, 510515 People’s Republic of China

**Keywords:** Cancer, Biomarkers, Oncology

## Abstract

The research on targeted therapy of hypopharyngeal cancer is very scarce. The discovery of new targeted driver genes will promote the progress of hypopharyngeal cancer therapy to a great extent. In our research, whole-exome sequencing in 10 patients with hypopharyngeal cancer was performed to identify single nucleotide variations (SNVs) and insertions and deletions (INDELs). American College of Medical Genetics and Genomics (ACMG) guidelines were used to evaluate the pathogenicity of the selected variants. 8113 mutation sites in 5326 genes were identified after strict screening. We identified 72 pathogenic mutations in 53 genes according to the ACMG guidelines. Gene Ontology (GO) annotation and KEGG enrichment analysis show the effect of these genes on cancer. Protein–protein interaction (PPI) was analyzed by string online software. The validation results of the ualcan database showed that 22 of the 53 genes may be related to the poor prognosis of patients with hypopharyngeal cancer. RBM20 has the most significant correlation with hypopharyngeal cancer, and it is likely to be the driver gene of hypopharyngeal cancer. In conclusion, we found possible therapeutic targets for hypopharyngeal cancer, especially RBM20 and KMT2C. Our study provides a basis for the pathogenesis and targeted therapy of hypopharyngeal cancer.

## Introduction

Hypopharyngeal cancer is the fourth most common cancer of the head and neck. Most patients with hypopharyngeal cancer have a history of smoking and drinking^1^. Squamous cell carcinoma accounts for the vast majority of pathological types of hypopharyngeal cancer, which is characterized by diffuse primary tumor with local dissemination of mucosa and submucosa^2^. Compared with other cancers, such as liver cancer, lung cancer, and other cancers with a higher incidence, there are currently fewer studies on hypopharyngeal cancer. Some of the biomarkers discovered have not yet been put into practice, and the oncogenes is relatively unclear. Therefore, it is particularly important to find more oncogenes related to the pathogenesis and progress of hypopharyngeal cancer, which will bring benefits for the early diagnosis and clinical treatment of hypopharyngeal cancer. Patients with hypopharyngeal cancer are often accompanied by lymph node metastasis at the time of treatment, which makes it difficult to be treated at present. The current treatment for hypopharyngeal cancer mainly includes surgery, immunotherapy, radiotherapy, chemotherapy, and combined therapy, but the efficacy is not satisfactory, and the five-year survival rate of patients is very low^3,4^. Therefore, early diagnosis is particularly important for patients with hypopharyngeal cancer.

In recent years, Next Generation Sequencing (NGS) has brought new breakthroughs to cancer in terms of formulating new cancer biomarkers and discovering mutations^5,6^. It mainly includes whole-genome sequencing (WGS) and whole-exome sequencing (WES). Since exons only account for about 1–2% of the whole genome and exon mutations are largely related to disease characteristics, WES has higher economic benefit compared with whole-genome sequencing^7,8^. Whole-exome sequencing plays an important role in identifying gene mutations. Bala et al. found a new tumor suppressor ARID2 in early-onset sporadic rectal cancer through whole-exome sequencing. Compared with patients without ARID2 alteration, patients with ARID2 alterations have poorer survival^9^. In addition, WES has brought new enlightenment for the development of patient treatment plans. PIK3CA and ERBB2 mutations were found in a whole exome sequencing study of cervical carcinomas. The combination of her2 inhibitor neratinib and PIK3CA inhibitor copanlisib has a better tumor regression effect than single inhibitor therapy^10^. Iyer et al. found that KRAS (G12V) mutations may hinder anti-EGFR therapy for gallbladder cancer patients through whole-exome sequencing studies^11^. Plenty of canonical cancer genes have been reported to be related to the development and prognosis of hypopharyngeal cancer in previous studies. These genes are also reported in TCGA and other databases, such as TP53, RAF-1, FHIT, etc.^12–14^, but there are still very few studies on hypopharyngeal cancer-related oncogenes, hence further exploration of hypopharyngeal oncogene is urgently needed.

In our research, we performed whole-exome sequencing in ten patients with hypopharyngeal cancer rather than targeted sequencing of specific genes. The sequenced data were compared with the reported genes in databases such as cosmic and TCGA, and some mutations that have not been reported yet were found. In addition, we compared and verified some classic oncogenes reported in the database with the mutations of ten patients. Finally, we used the ualcan database to further verify the gene expression level and its relationship with prognosis. This study provides evidence for studying the gene mutation information of liver cancer.

## Results

### Patient characteristics

The clinicopathological data of 10 hypopharyngeal cancer patients are summarized in Table 1. The collected clinical data mainly include age, clinical stage, tumor diameter, lymphatic metastasis, distant metastasis, tumor differentiation degree, etc.

**Table 1:** The clinicopathological data of 10 hypopharyngeal carcinoma patients.

Variable	No of patients N = 10 (%)
**Age (years)**	
Mean ± SD	55.1 ± 9.42
Range	35–68
**Clinical stage**	
I	0 (0)
II	0 (0)
III	8 (80)
IV	2 (20)
**Tumor size**	
T1	0 (0)
T2	5 (50)
T3	2 (20)
T4	3 (30)
**Lymph nodes status**	
N0	0 (0)
N1	5 (50)
N2	4 (40)
N3	1 (10)
NA	2 (20)
**Distant metastasis**	
M0	8 (80)
M1	2 (20)
**Tumor differentiation**	
Low	3 (30)
Medium	6 (60)
High	1 (10)

### Gene variation spectrum

**Figure 1:**
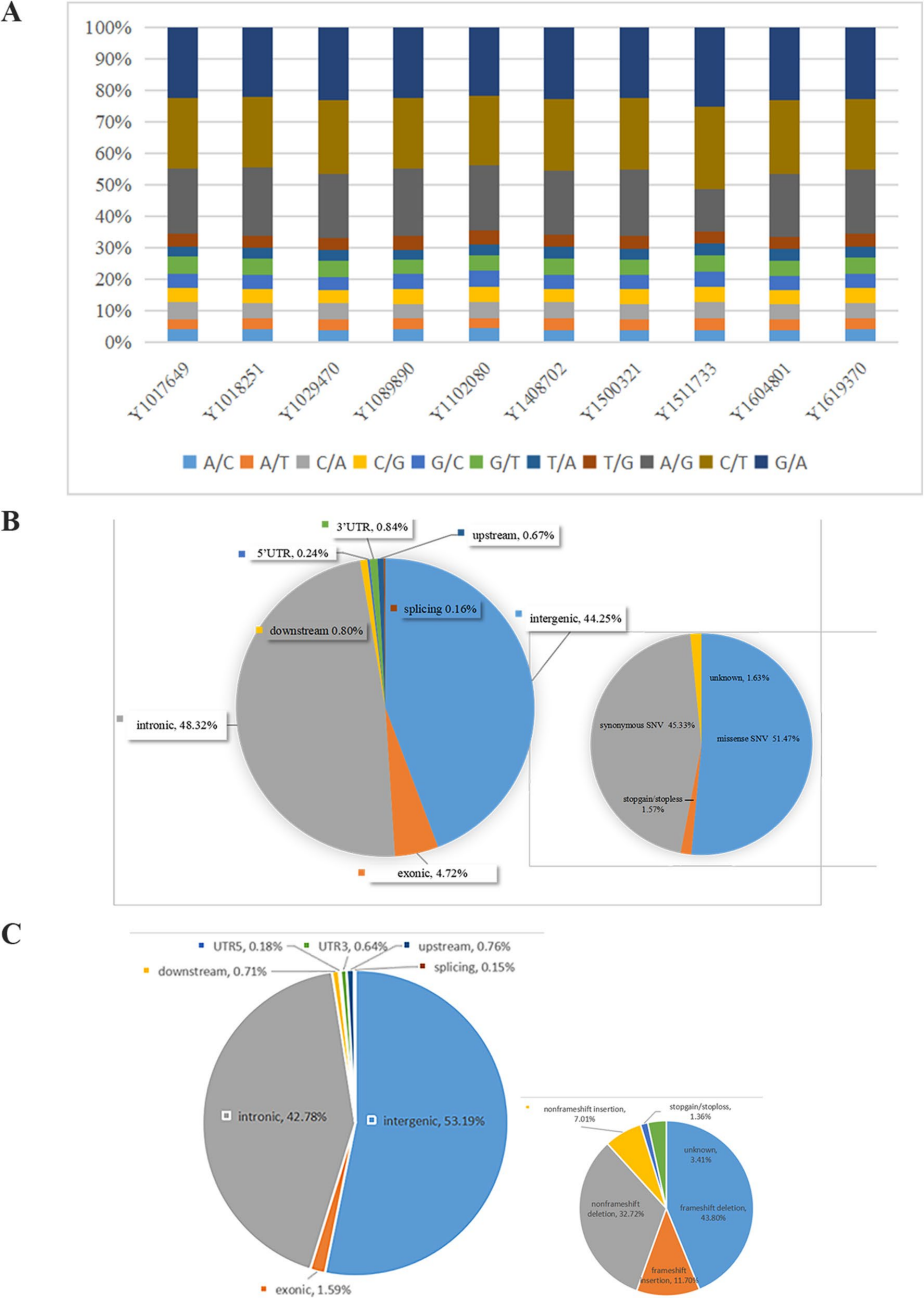
Targeted sequencing of 10 patients with hypopharyngeal cancer (**A**). The proportion of nucleotide mutations in 10 patients with hypopharyngeal cancer (**B**). The proportion of mutation in different regions in SNV and the proportion of different mutation type in exon regions (**C**). The proportion of mutation in different regions in indel and the proportion of different mutation type in exon regions.

The detection of SNV and indel in 10 patients with hypopharyngeal cancer was statistically analyzed. We found that A/G, C/T, G/A transitions were more common than other types of single nucleotide mutations in all patients with hypopharyngeal cancer (Fig. 1A). Exon mutations accounted for 4.72% of all mutations. The mutation types of single nucleotide mutations in the exon region were counted. The results showed that missense mutations account for 51.47% of all exon mutations. Stop gain/loss mutations account for 1.57% of all mutations, and nonsense mutations account for 45.33% of all mutations. In addition, there are 1.63% of unknown mutations (Fig. 1B). In the detection of insertions and deletions, exon mutations accounted for 1.59%. Frameshift deletion and frameshift insertion accounted for 43.8% and 11.7% of all exon mutations, respectively. Nonframeshift deletion and nonframeshift insertion accounted for 32.72% and 7.01% of all exon mutations, and stop gain/loss accounted for 1.36% of all mutations. In addition, there are 3.41% of unknown mutations (Fig. 1C). In order to better understand the genetic mutations of each patient, the number of SNP/indel in different regions of the genome of each patient and the distribution of the number of different types of SNP/indel in the coding region were counted (Supplementary Figs. 1, 2).

We identified 8113 non-synonymous mutations after screening, including 8096 missense mutations, 1 stop gain mutation and 16 unknown mutations in 5326 genes. There were 1066 mutated genes in two patients, 339 in three patients, 80 in four patients and 22 in five patients. Interestingly, 8 genes including MEGF8, ITPR1, DYSF, DNAH10, CUL7, MYH14, LRP1, and ASTN1 have mutations in six patients, 3 genes including TTN, ASH1L, and MYH11 have mutations in seven patients, and KMT2C has mutations in ten patients. We found that all of the 12 genes had new mutations except kmt2c by comparing the dbSNP database (Table 2, Supplementary Tables 1, 2).

**Table 2:** Mutations of mutated genes in at least 6 patients.

Gene	Mutation proportion (%)	Mutation information
MEGF8	60	rs377748543, rs370522595, 3 novel mutations
ITPR1	60	rs752791333, rs773763162, 3 novel mutation
DYSF	60	rs573666770, 4 novel mutations
DNAH10	60	rs148844278, rs748343428, 2 novel mutations
CUL7	60	rs757730802, rs373305024, 3 novel mutations
TTN	70	rs372496072, rs35683768, rs878903962, rs373854384, rs371908649, 3 novel mutations
MYH14	60	rs762779652, rs140118363, 4 novel mutations
LRP1	60	rs199726731, 9 novel mutations
ASTN1	60	6 novel mutations
ASH1L	70	6 novel mutations
MYH11	70	rs751495086, rs757099566, 4 novel mutations
KMT2C	100	rs2479172, rs28522267, rs77735469, rs201062304, rs28522267

### High-frequency mutation genes in hypopharyngeal cancer in TCGA database

The 20 oncogenes most frequently mutated in the TCGA database were compared with our screened data, and the results showed that 13 genes including TTN, TP53, ANK3, UPF2, C6, BRCA2, CD163L1, ZNF831, KRT85, MACF1, SYT6, TPO, and SLIT2 had mutations in our samples (Table 3). TTN (70%), ANK3 (40%), and TP53 (30%) have a higher mutation rate, which is also ranked in the top three in the TCGA database. It showed that our results are consistent with the results of the TCGA database.

**Table 3:** Comparison of the TOP20 genes of hypopharyngeal carcinoma in the TCGA database and the samples in this research.

	1	2	3	4	5	6	7	8	9	10	Mutation proportion (%)
TTN	−	+	+	−	+	+	+	−	+	+	70
TP53	−	−	+	−	+	−	−	+	−	−	30
ANK3	+	+	−	−	−	+	−	−	+	−	40
UPF2	−	+	−	−	−	−	−	−	−	−	10
MFAP3	−	−	−	−	−	−	−	−	−	−	0
DST	−	−	−	−	−	−	−	−	−	−	0
C6	−	+	−	−	−	−	−	−	−	−	10
BRCA2	−	+	−	−	−	−	−	−	−	−	10
CD163L1	−	−	+	−	−	−	−	−	−	−	10
MUC16	−	−	−	−	−	−	−	−	−	−	0
ZNF831	−	−	−	−	−	−	+	−	−	−	10
KRT85	−	−	−	−	−	−	−	−	+	−	10
MACF1	−	−	−	−	−	+	−	+	−	−	20
CCDC146	−	−	−	−	−	−	−	−	−	−	0
SYT6	−	+	−	−	−	−	−	−	−	−	10
ANO7	−	−	−	−	−	−	−	−	−	−	0
TPO	−	−	−	−	−	−	−	−	+	−	10
RBAK-RB	−	−	−	−	−	−	−	−	−	−	0
GRM8	−	−	−	−	−	−	−	−	−	−	0
SLIT2	+	–	−	−	−	−	−	−	+	−	20

+ mutated in the sample, − not mutated in the sample.

### Screening pathogenic mutations associated with hypopharyngeal carcinoma based on ACMG guidelines

72 pathogenic or possibly pathogenic mutations were identified in 53 genes according to the ACMG guidelines, including SNVs or INDELs (Supplementary Table 3). There were 2 pathogenic or possibly pathogenic mutations in BIVM-ERCC5, FBN2, MYH11, SCN2A, S4CNA and SDHA, 3 pathogenic or possibly pathogenic mutations in RYR1 and SCN5A, and 4 pathogenic or possibly pathogenic mutations in LDLR, TP53 and TTN. 4 unreported mutations were found by comparison to the dbSNP database, and these mutations may cause disease, including two mutations in BIVM-ERCC5 (exon6:c.C640T:p.R214C), (exon14:c. C2002T:p.R668C), GJA3 (exon2:c.C56T:p.T19M), SPG7 (exon9:c.C1198T:p.R400W), these mutations may be related to the pathogenesis of hypopharyngeal cancer.

### GO annotation of driver genes associated with hypopharyngeal carcinoma

**Figure 2:**
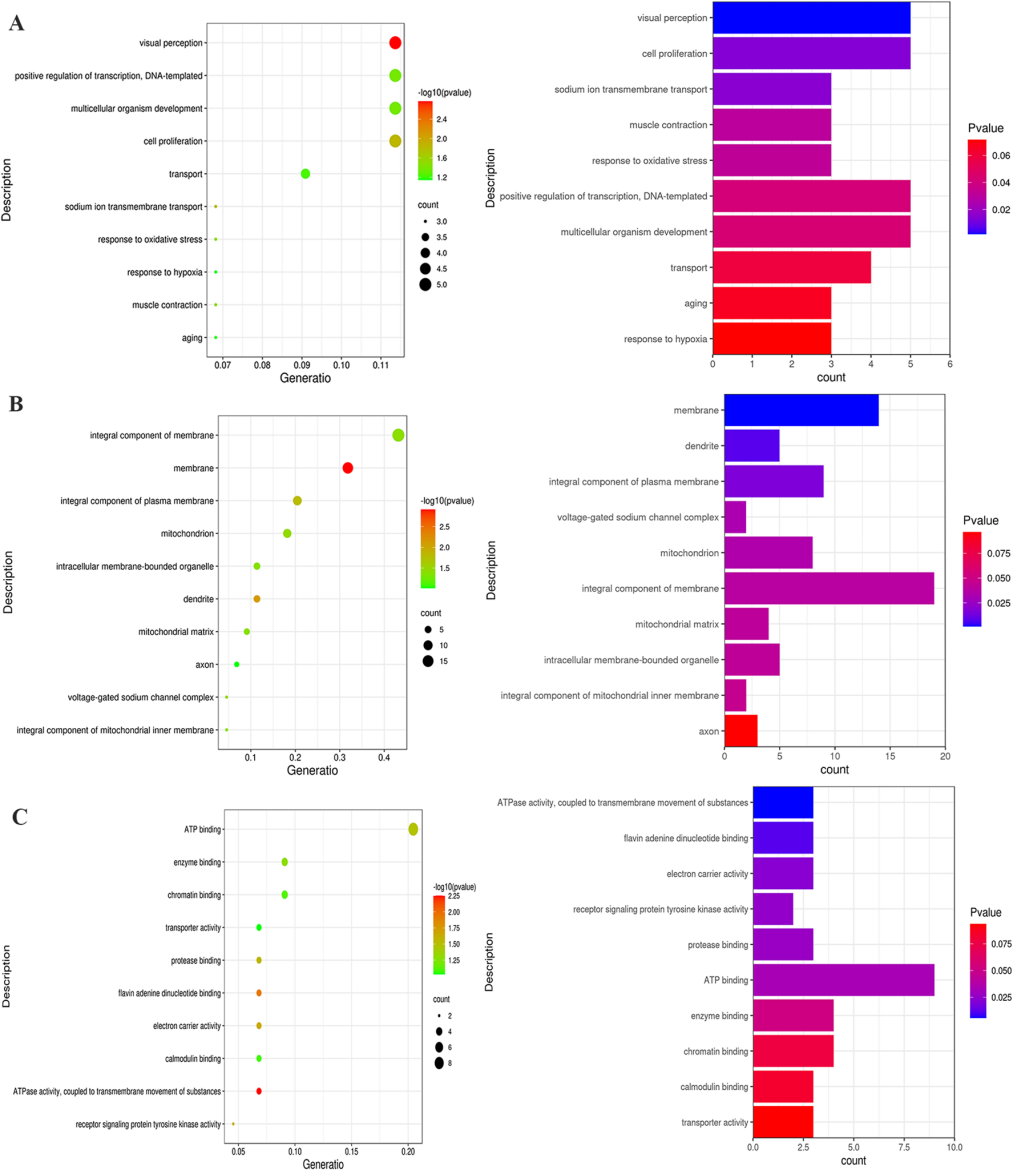
TOP 10 in Go enrichment analysis (**A**). Biological process (GO-BP) of 53 driver genes (**B**). Cellular component (GO-CC) of driver genes (**C**). Molecular function (GO-MF) of driver genes.

Gene ontology annotation and pathway analyses were performed on 53 driver genes and possibly driver genes. The BP of these genes is related to muscle contraction, visual perception, cell proliferation, positive regulation of transcription, DNA-templated, multicellular organism development, sodium ion transmembrane transport, positive regulation of gene expression, nervous system development, transport, etc. The main cellular components of these genes involve integral component of membrane, integral component of plasma membrane, mitochondrion, dendrite, intracellular membrane-bounded organelle, Z disc, mitochondrial matrix, voltage-gated sodium channel complex, apical part of cell, etc. GP-MF annotation showed that these genes are related to some molecular functions, including protein binding, ATP binding, calcium ion binding, calmodulin binding, enzyme binding, protein binding, ubiquitin protein ligase binding, voltage-gated sodium channel activity ATPase activity, coupled to transmembrane movement of substances, flavin adenine dinucleotide binding (Fig. 2, Supplementary Table 4).

### Altered pathways

**Figure 3:**
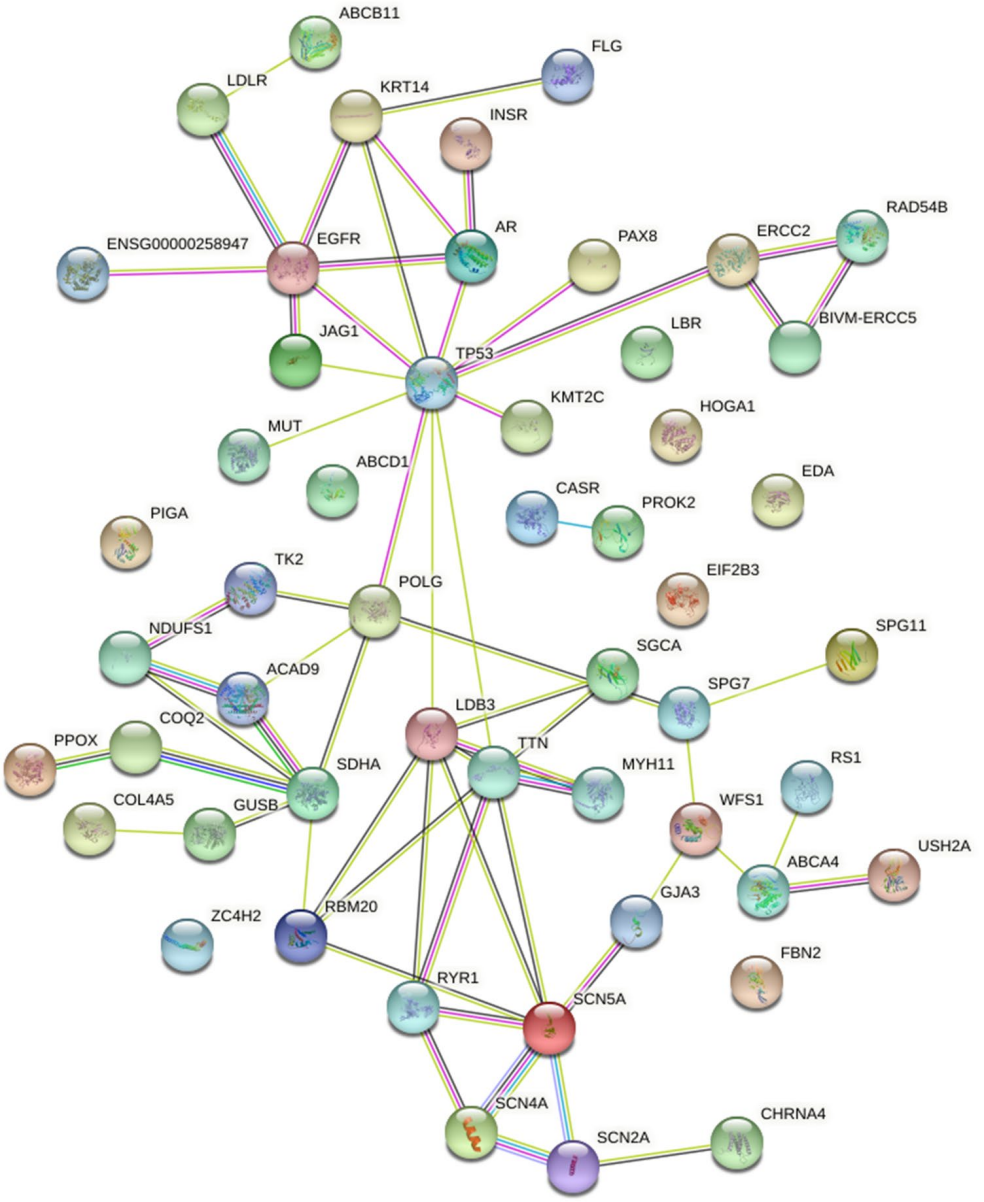
PPI network of 53 driver genes.

53 driver genes associated with hypopharyngeal carcinoma were analyzed by KEGG enrichment (https://www.kegg.jp/kegg/), and the results showed that these genes were highly enriched in some cancers and cancer-related pathways (Table 4). Enrichment pathways mainly include: (a) Various cancers, including thyroid cancer, bladder cancer, endometrial cancer, non-small cell lung cancer, melanoma, pancreatic cancer, colorectal cancer, small cell lung cancer, etc. (b) some signaling pathways closely related to cancer, such as MAPK signaling pathway, HIF-1 signaling pathway, central carbon metabolism in cancer, etc. We constructed a PPI network of 53 driver genes to understand the interaction between 53 driver genes (Fig. 3). The figure shows that these genes include 52 nodes and 62 edges.

**Table 4:** Pathway annotation of 53 driver genes.

ID	Description	Background number	P-value	count	Gene ID
hsa01100	Metabolic pathways	1433	1.21 × 10^–4^	9	PIGA|TK2|HOGA1|GUSB |NDUFS1|COQ2|PPOX|MUT|SDHA
hsa05200	Pathways in cancer	530	8.35 × 10^–5^	6	COL4A5|TP53|JAG1|EGFR|PAX8|AR
hsa01522	Endocrine resistance	98	1.14 × 10^–5^	4	TP53|ABCB11|EGFR|JAG1
hsa05165	Human papillomavirus infection	330	1.08 × 10^–3^	4	TP53|JAG1|COL4A5|EGFR
hsa04151	PI3K-Akt signaling pathway	354	1.40 × 10^–3^	4	TP53|COL4A5|EGFR|INSR
hsa02010	ABC transporters	45	3.84 × 10^–5^	3	ABCA4|ABCB11|ABCD1
hsa03420	Nucleotide excision repair	47	4.34 × 10^–5^	3	ERCC2|ERCC5|BIVM-ERCC5
hsa05215	Prostate cancer	97	3.41 × 10^–4^	3	TP53|AR|EGFR
hsa05224	Breast cancer	147	1.11 × 10^–3^	3	TP53|JAG1|EGFR
hsa04932	Non-alcoholic fatty liver disease (NAFLD)	149	1.15 × 10^–3^	3	INSR|SDHA|NDUFS1
hsa05160	Hepatitis C	155	1.29 × 10^–3^	3	TP53|LDLR|EGFR
hsa05016	Huntington disease	193	2.38 × 10^–3^	3	TP53|SDHA|NDUFS1
hsa04010	MAPK signaling pathway	295	765 × 10^–3^	3	TP53|EGFR|INSR
hsa00630	Glyoxylate and dicarboxylate metabolism	30	8.63 × 10^–4^	2	HOGA1|MUT
hsa05216	Thyroid cancer	37	1.28 × 10^–3^	2	TP53|PAX8
hsa05219	Bladder cancer	41	1.56 × 10^–3^	2	TP53|EGFR
hsa00860	Porphyrin and chlorophyll metabolism	42	1.63 × 10^–3^	2	GUSB|PPOX
hsa04913	Ovarian steroidogenesis	49	2.18 × 10^–3^	2	LDLR|INSR
hsa04979	Cholesterol metabolism	50	2.27 × 10^–3^	2	ABCB11|LDLR
hsa05213	Endometrial cancer	58	3.00 × 10^–3^	2	TP53|EGFR
hsa05223	Non-small cell lung cancer	66	3.84 × 10^–3^	2	TP53|EGFR
hsa05230	Central carbon metabolism in cancer	69	4.18 × 10^–3^	2	TP53|EGFR
hsa04520	Adherens junction	72	4.53 × 10^–3^	2	EGFR|INSR
hsa04976	Bile secretion	72	4.53 × 10^–3^	2	ABCB11|LDLR
hsa05218	Melanoma	72	4.53 × 10^–3^	2	TP53|EGFR
hsa05212	Pancreatic cancer	75	4.89 × 10^–3^	2	TP53|EGFR
hsa05214	Glioma	75	4.89 × 10^–3^	2	TP53|EGFR
hsa00983	Drug metabolism—other enzymes	79	5.40 × 10^–3^	2	GUSB|TK2
hsa05210	Colorectal cancer	86	6.34 × 10^–3^	2	TP53|EGFR
hsa04540	Gap junction	88	6.63 × 10^–3^	2	EGFR|TUBB3
hsa04211	Longevity regulating pathway	89	6.77 × 10^–3^	2	TP53|INSR
hsa05410	Hypertrophic cardiomyopathy (HCM)	90	6.91 × 10^–3^	2	TTN|SGCA
hsa05222	Small cell lung cancer	93	7.35 × 10^–3^	2	TP53|COL4A5
hsa05414	Dilated cardiomyopathy (DCM)	96	7.81 × 10^–3^	2	TTN|SGCA
hsa04928	Parathyroid hormone synthesis, secretion and action	106	9.41 × 10^–3^	2	CASR|EGFR
hsa04066	HIF-1 signaling pathway	109	9.92 × 10^–3^	2	EGFR|INSR

### The gene expression level and its relationship with prognosis were further verified by UALCAN database

**Figure 4:**
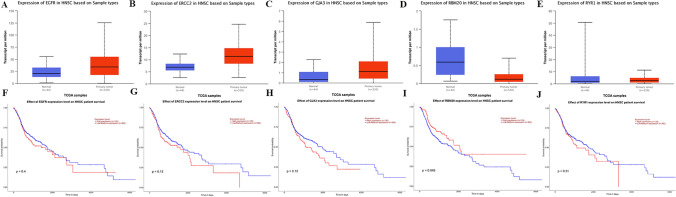
Survival curve and expression of EGFR, ERCC2, GJA3, RBM20 and RYR1 (UALCAN database). (**A–E**) Survival curves. (**F–J**) Expression of genes in liver cancer and normal liver tissues.

To further confirm the relationship between the screened genes and hypopharyngeal cancer, we verified 53 driver genes using the UALCAN database. We found that 32 genes were highly expressed in hypopharyngeal cancer, and the high expression of 16 genes was associated with poor OS, including ACAD9, ERCC5, COL4A5, COQ2, EGFR, EIF2B3, ERCC2, GJA3, JAG1, LDLR, POLG, PROK2, RAD54B, RYR1, SDHA and TUBB3. 18 genes were low-expressed in the hypopharyngeal cancer tissue, and the low expression of 6 genes was associated with the poor OS of the patient, including ABCB11, AR, FLG, MUT, RBM20 and SPG11. These 22 genes may be the genes that lead to poor prognosis in patients with hypopharyngeal cancer. Figure 4 shows the five genes with the highest significance in the survival curve (Fig. 4), in which RBM20 shows the most significant correlation with hypopharyngeal cancer, and its expression in tumor tissues is much lower than that in normal tissues. The OS of patients with low expression is significantly lower than that of patients with high expression (P = 0.045). After whole exome sequencing of 10 patients with hypopharyngeal cancer, we found that there were two mutations in the exon region of RBM20 that may lead to pathogenicity (Exon2: c.c1138t: p.r380w), (exon9:c.C1913T:p.P638L).

## Discussion

Hypopharyngeal cancer is relatively rare compared with other cancers, accounting for about 3% of head and neck malignant tumors, and most patients are already in the advanced stage when they are diagnosed^15,16^. At present, there are few studies on the mechanism of hypopharyngeal cancer. In order to better treat hypopharyngeal cancer, ten patients with hypopharyngeal cancer were subjected to whole-exome sequencing rather than targeted sequencing of specific genes, aiming to discover more mutations related to the occurrence and development of hypopharyngeal cancer.

8113 mutation sites were found in 5326 genes after strict screening conditions. And we found that MEGF8, ITPR1, DYSF, DNAH10, CUL7, MYH14, LRP1, ASTN1, TTN, ASH1L, and MYH11 mutated in at least 6 patients, while KMT2C mutated in 10 patients. To verify the accuracy of our results in this study, our screened data were compared with the TOP20 gene in the TCGA database, and we found that the top three genes (TTN, ANK3, and TP53) in the hypopharyngeal cancer mutation genes in the TCGA database also had mutations in more patients in our samples. Moreover, TP53 mutation has been found to indicate a worse prognosis in patients with hypopharyngeal squamous cell carcinoma in previous studies, which to some extent proved the accuracy of our results^17^. Wu et al. explored the driver gene in hypopharyngeal cancer using Whole-exome sequencing and identified some novel mutations in 2017^18^, but it is still completely insufficient to make up for the gap in the research on the driving gene of hypopharyngeal cancer, hence the need for further exploration inquiry. We identified a great number of novel mutations that have not been reported which may be related to the pathogenesis of hypopharyngeal cancer.

In order to determine the pathogenicity of the mutations, 72 mutations in 53 genes were selected according to the international ACMG guidelines. We found that two pathogenic or possibly pathogenic mutation sites in BIVM-ERCC5, FBN2, MYH11, SCN2A, S4CNA and SDHA, three pathogenic or possibly pathogenic mutation sites in RYR1 and SCN5A, and four pathogenic or possibly pathogenic mutation sites in LDLR, TP53 and TTN. In addition, we found four sites not reported in dbSNP database, including two mutations in BIVM-ERCC5 (exon6: c.c640t: p.r214c), (exon14: c.c2002t: p.r668c), GJA3 (Exon2: c.c56t: p.t19m) and SPG7 (exon9: c.c1198t: p.r400w), which may be related to the occurrence and development of hypopharyngeal cancer.

To further confirm the role of these causative genes and whether they are associated with the pathogenesis of hypopharyngeal cancer, GO annotation, KEGG enrichment analysis, and PPI network were constructed. The results show that many of these genes are associated with cancer. However, hypopharyngeal cancer is a small cancer species, so there are few reports about hypopharyngeal cancer in the database, which is also one of the significance of our study. Interestingly, we found that TTN, MYH11, SDHA, and RYR1 have mutations in many samples, and they have more than one disease-causing mutation in 10 patients.

KMT2C, a member of histone methyltransferase (H3K4ME3), is a kind of chromotin modifying and remodelling protein^19^. KMT2C can catalyze the methylation of protein sites to change the structure of chromosomes and finally affect the transcription process of target genes^20,21^. Previous studies have shown that KMT2C is mutated in a variety of cancers, including osteosarcoma, acute myeloid leukemia, breast cancer, and gastric cancer^22–25^. However, there are very few reports about the relationship between KMT2C mutation and hypopharyngeal cancer. In our research, GO-MF results showed that KMT2C was involved in protein binding, we found that all samples had KMT2C mutation after strict screening of the whole-exome sequencing results of ten patients, which may indicate the relationship between KMT2C and the pathogenesis of hypopharyngeal cancer, and provide thinking for future research of the pathogenesis of hypopharyngeal cancer.

The protein encoded by MYH11 is a smooth muscle myosin belonging to the myosin heavy chain family, which acts as a contractile protein by participating in the hydrolysis of adenosine triphosphate^26^. There have been some previous studies on the relationship between MYH11 and cancer. Studies have confirmed that it is related to the pathogenesis or prognosis of lung cancer, acute myeloid leukemia, gastric cancer, colorectal cancer and breast cancer^26–29^. However, the relationship between MYH11 and hypopharyngeal cancer has not yet been reported. In our research, we found that MYH11 mutated in 7 patients, and we found two pathogenic or possibly pathogenic mutations (rs375159635, rs751495086), which may be related to the pathogenesis of hypopharyngeal cancer. In addition, SDHA is mainly related to gangliomas^30–32^, RYR1 is mainly related to myopathy^33,34^, and TTN is mainly related to dilated cardiomyopathy^35,36^. Interestingly, we found that these three genes have mutations in more than five patients, and all of them have two or more pathogenic or possibly pathogenic mutation sites, which indicates their potential in the pathogenesis of hypopharyngeal cancer.

The protein encoded by RBM20 can bind to RNA and regulate splicing. At present, it is considered to be related to various cardiomyopathy. After verifying the selected 53 driver genes using the UALCAN database, we found that RBM20 was highly expressed in the hypopharyngeal cancer tissue. The high expression of RBM20 predicts a worse OS in patients with hypopharyngeal cancer, indicating that RBM20 is likely to be related to the pathogenesis or disease progression of hypopharyngeal cancer.

## Conclusion

Inevitably, there are shortcomings in this study. Above all, our sample size is very small, and the conclusion is not reliable enough. Besides, although we have performed whole exome sequencing and a series of analysis, more experimental verification is needed.

We performed whole-exome sequencing on 10 patients with hypopharyngeal cancer and screened out some genes that may be related to the pathogenesis of hypopharyngeal cancer, including a great number of novel mutations that have not been reported, especially mutations in RBM20 and KMT2C. In our samples, KMT2C, which participates in protein binding, has pathogenic mutations in all samples, and the expression of RBM20 is related to the survival of patients with hypopharyngeal cancer, indicating that they are likely to be related to the pathogenesis of hypopharyngeal cancer. However, more adequate descriptive and functional studies are required to fully reveal the pathogenic roles of RBM20 and KMT2C in the pathophysiology of hypopharyngeal cancer. Our research has deepened the understanding of the pathogenesis of hypopharyngeal cancer and provided a foundation for subsequent research.

## Materials and methods

### Study population

10 patients who received surgical treatment in the Affiliated Nanhua Hospital, University of South China from 2016 to 2020 were included in this study. There was no blood relationship between the patients. Patients who had previously received systematic treatment or suffered from hypopharyngeal cancer combined with other tumors were excluded. Ten patients with hypopharyngeal cancer were confirmed by pathological biopsy. Fresh tissue samples were collected from the tumor tissue center. The sequencing samples were quickly frozen in liquid nitrogen and transferred to the – 80 °C refrigerator for preservation. Slice samples were stored in 10% neutral formalin. Our research was approved by the Ethics Committee of the University of South China and complies with the Declaration of Helsinki. All patients agreed to this study and signed an informed consent form.

### DNA extraction and gene sequencing

Qubit 2.0 is used to accurately quantify the concentration of DNA samples. DNA samples with a DNA concentration of ≥ 20 ng/µL and a total amount of 0.6 µg or more are used to build the library. Genomic DNA was randomly fragmented into 180–280 bp fragments using a Covaris fragmentation apparatus. The Agilent sureselect human all exon V5/v6 kit was used for the construction and capture of genomic DNA library. The library with a specific index was hybridized with biotin labeled probe in the liquid phase. Magnetic beads with streptomycin were used to capture the exons, which were linearly amplified by PCR for library quality inspection. Qubit 2.0, Q-PCR, and Agilent 2100 were used to quantify and detect the library.

### Sequencing data filtering

To ensure the quality of information analysis, raw reads are finely filtered to obtain clean reads. The steps of data processing include: (a) removing reads with adapters; (b) Reads in which the proportion of N more than 10% is removed (N indicates that the nucleobase information cannot be determined); (c) When the number of low-quality (less than 5) bases contained in the single-ended sequencing read exceeds 50% of the length proportion of the read strip, the pair of paired reads are removed. For those data using double-ended sequencing, we required an average Q30 ratio of above 80% and an average error rate of below 0.1%.

### Data analysis

The effective sequencing data were compared to the reference genome (human genome) by BWA and samblaster_ B37), and then samblaster was used to mark repeated reads to get the final comparison results. Samtools is used to detect and filter SNP and indel mutations. Annovar is used to annotate the structure and function of the detected variation. We used the annotated and visual database (David) bioinformatics resources 6.8 to identify the biological processes and pathways that 10 patients significantly changed. GO terminology is mainly annotated from GO-CC (cell component), GO-MF (molecular function) and GO-BP (biological process). KEGG pathway database is used for pathway enrichment (https://www.kegg.jp/kegg/)^37,38^. P < 0.05 was considered to be statistically significant in the Go annotation and KEGG enrichment analysis. String online software (https://string-db.org/) was used to predict protein–protein interaction (PPI). The UALCAN database was used to further verify the level of gene expression and its relationship with prognosis.

### Statement of ethics

Our study was approved by the ethics committee of Affiliated Nanhua Hospital, University of South China (approval No. 202008). Informed consent was obtained from all individual participants in the study.

## Supplementary Information

Supplementary Tables.

Supplementary Figures.

## Data Availability

The original sequencing data has been uploaded to the NCBI database (prjna846060, https://www.ncbi.nlm.nih.gov/bioproject/PRJNA846060.
