# Supplementary material for: Application value of whole exome sequencing in screening and identifying novel mutations of hypopharyngeal cancer
Source: Sci Rep. 2023 Jan 3;13:107. doi: 10.1038/s41598-022-27273-w (PMC9810646; doi:10.1038/s41598-022-27273-w)
Supplement: Supplementary file 1 — Supplementary Tables. [file 41598_2022_27273_MOESM1_ESM.docx]

**Table F1** Mutations of mutated genes in at least 6 patients

| **Gene** | **Mutation proportion** | **Mutation information** |
| --- | --- | --- |
| **MEGF8** | 6 | p.R2617W p.R2684W(rs377748543),p.G408V(a novel mutation), p.R1037W p.R1104W(rs370522595)  p.G1279R p.G1346R(a novel mutation),p.S769F p.S836F(a novel mutation) |
| **ITPR1** | 6 | p.R1080W p.R1071W p.R1086W(rs752791333), p.Q1308R p.Q1299R p.Q1314R(a novel mutation), p.M754K p.M769K(a novel mutation), p.L1920F p.L1935F p.L1968F(a novel mutation), p.R2489Q p.R2504Q p.R2537Q(rs773763162) |
| **DYSF** | 6 | p.F853Y p.F854Y(a novel mutation),p.L768Q :p.L769Q(a novel mutation), p.D1133Y p.D1134Y(a novel mutation),p.R794L p.R795L(a novel mutation), p.A1319T p.A1320T(rs573666770) |
| **DNAH10** | 6 | p.R2793H(rs148844278),p.R2793H(rs148844278),p.E676K(a novel mutation), p.L4149H(a novel mutation),p.R2218H(rs748343428), |
| **CUL7** | 6 | p.R1103C p.R1019C(rs757730802), p.P1385L p.P1301L(a novel mutation), p.Q810H p.Q726H(a novel mutation),p.L169H p.L117H(a novel mutation), p.R503C p.R419C(rs373305024) |
| **TTN** | 7 | p.R13752Q p.R13877Q(rs372496072),  p.P23731S p.P23856S(a novel mutation), p.D60Y(rs35683768),p.A189D(a novel mutation), p.D25491N p.D25683N(rs878903962),rs373854384, rs371908649,p.T21272I p.T21464I(a novel mutation) |
| **MYH14** | 6 | p.D539N p.D547N(rs762779652),p.I807T p.I848T(a novel mutation), p.E1249K p.E1257K(a novel mutation),p.L1474R p.L1482R(a novel mutation), p.R1521Q p.R1529Q(rs140118363),p.R126Q(a novel mutation) |
| **LRP1** | 6 | p.R1497Q(a novel mutation),p.G2487S(rs199726731), p.R3040L(a novel mutation),p.E3485Q(a novel mutation), p.R350H(a novel mutation),p.T3335M(a novel mutation), p.L1568F(a novel mutation),p.T2303M(a novel mutation), p.R3879C(a novel mutation),p.C3638S(a novel mutation) |
| **ASTN1** | 6 | p.K65N(a novel mutation),p.G1051V(a novel mutation), p.G1170W(a novel mutation),p.Q190R(a novel mutation), p.S1080I(a novel mutation),p.R1254H(a novel mutation) |
| **ASH1L** | 7 | p.R2738W(a novel mutation),p.P2335L(a novel mutation), p.R1840W(a novel mutation),p.D2050Y(a novel mutation), p.P1423S(a novel mutation),p.R1053H(a novel mutation) |
| **MYH11** | 7 | p.R439C p.R446C(a novel mutation),p.E1679K p.E1686K(a novel mutation), p.T413I p.T420I(a novel mutation),p.R1792Q p.R1799Q(rs751495086),  p.E1920K p.E1927K(rs757099566),p.V550M p.V557M(a novel mutation) |
| KMT2C | 10 | p.G838S(rs2479172),p.G838S(rs2479172),p.G838S(rs2479172), p.C988F(rs28522267),p.G838S(rs2479172),p.C988F(rs28522267), p.Y987H(rs77735469),p.G838S(rs2479172),p.G838S(rs2479172), p.Y987H(rs77735469),p.G838S(rs2479172),p.V3102L(rs201062304), p.C988F(rs28522267),p.Y987H(rs77735469), p.G838S(rs2479172),p.G838S(rs2479172) |

**Table F2** Mutations of mutated genes in at least 2 patients

|  | ZZEF1 | ZSWIM8 | ZSWIM6 | ZSCAN30 | ZRANB1 | ZNF804B | ZNF786 | ZNF711 | ZNF704 | ZNF609 |
| --- | --- | --- | --- | --- | --- | --- | --- | --- | --- | --- |
| **patients  with mutations(n)** | 2 | 2 | 2 | 3 | 3 | 2 | 2 | 2 | 2 | 3 |
|  | ZNF583 | ZNF574 | ZNF462 | ZNF423 | ZNF398 | ZNF277 | ZNF236 | ZNF217 | ZNF205 | ZNF142 |
| **patients  with mutations(n)** | 2 | 2 | 3 | 2 | 2 | 2 | 2 | 2 | 2 | 5 |
|  | ZNF112 | ZMYND8 | ZMYM3 | ZMIZ2 | ZMIZ1 | ZHX1 | ZFYVE28 | ZFYVE1 | ZFP64 | ZFP37 |
| **patients  with mutations(n)** | 2 | 2 | 2 | 3 | 3 | 3 | 2 | 3 | 2 | 2 |
|  | ZFHX4 | ZFC3H1 | ZEB2 | ZCCHC6 | ZCCHC11 | ZC3H13 | ZBTB40 | ZBTB4 | ZBTB20 | ZBTB18 |
| **patients  with mutations(n)** | 5 | 2 | 2 | 2 | 2 | 3 | 2 | 2 | 3 | 4 |
|  | YIF1A | YARS | XYLT2 | XPO1 | XKR6 | XIRP2 | XAB2 | WWP2 | WWC1 | WRN |
| **patients  with mutations(n)** | 4 | 2 | 2 | 2 | 2 | 3 | 2 | 2 | 2 | 3 |
|  | WNK3 | WNK2 | WNK1 | WFS1 | WFIKKN2 | WDR96 | WDR72 | WDR7 | WDR62 | WDR6 |
| **patients  with mutations(n)** | 2 | 4 | 2 | 2 | 2 | 2 | 2 | 3 | 2 | 2 |
|  | WDR59 | WDR5 | WDR44 | WDR33 | WDR19 | WDHD1 | WDFY3 | VWF | VWCE | VPS8 |
| **patients  with mutations(n)** | 2 | 2 | 2 | 2 | 2 | 2 | 3 | 3 | 2 | 2 |
|  | VPS18 | VPS13C | VPS13B | VIL1 | VGLL4 | VCPIP1 | VCL | VCAN | UTRN | UTP6 |
| **patients  with mutations(n)** | 2 | 2 | 3 | 2 | 2 | 2 | 2 | 2 | 3 | 2 |
|  | UTP14A | USP7 | USP6NL | USP35 | USP34 | USP19 | UPF3B | UNCX | UNC5B | UNC13B |
| **patients  with mutations(n)** | 2 | 3 | 2 | 3 | 2 | 2 | 2 | 2 | 2 | 3 |
|  | UHRF1BP1L | UHRF1BP1 | UGT1A1 | UBR5 | UBR4 | UBR2 | UBR1 | UBP1 | UBN2 | UBE2O |
| **patients  with mutations(n)** | 2 | 2 | 2 | 2 | 3 | 2 | 2 | 2 | 2 | 2 |
|  | UBA7 | TYROBP | TYRO3 | TULP3 | TUBGCP6 | TTN | TTLL9 | TTLL12 | TTC3 | TTC29 |
| **patients  with mutations(n)** | 2 | 2 | 3 | 2 | 2 | 7 | 3 | 2 | 4 | 2 |
|  | TTC14 | TTBK2 | TTBK1 | TSR1 | TRRAP | TRPV3 | TRPV1 | TRPS1 | TRPM4 | TRPM2 |
| **patients  with mutations(n)** | 2 | 2 | 2 | 2 | 4 | 2 | 2 | 2 | 2 | 2 |
|  | TRIP12 | TRIO | TRIM67 | TRIM46 | TRIM37 | TRIM33 | TRIM28 | TRIM2 | TREH | TRAPPC9 |
| **patients  with mutations(n)** | 2 | 2 | 2 | 2 | 2 | 2 | 2 | 2 | 2 | 2 |
|  | TRAPPC10 | TRANK1 | TRABD | TPM2 | TPCN2 | TP53BP1 | TP53 | TOPORS | TOPBP1 | TOP2B |
| **patients  with mutations(n)** | 2 | 3 | 2 | 2 | 3 | 5 | 3 | 2 | 2 | 2 |
|  | TOP1 | TNS1 | TNRC6C | TNRC6B | TNPO2 | TNIK | TNC | TMX3 | TMTC2 | TMPRSS9 |
| **patients  with mutations(n)** | 2 | 3 | 2 | 2 | 2 | 2 | 2 | 2 | 2 | 2 |
|  | TMPRSS7 | TMPRSS15 | TMPRSS13 | TMEM8B | TMCC2 | TMCC1 | TMC3 | TMC2 | TLR9 | TLN1 |
| **patients  with mutations(n)** | 2 | 3 | 2 | 2 | 2 | 2 | 2 | 2 | 2 | 2 |
|  | TLL1 | TLE4 | TLE2 | TJP1 | TIE1 | TICRR | THSD7B | THSD7A | THNSL2 | THBS4 |
| **patients  with mutations(n)** | 2 | 2 | 2 | 3 | 3 | 2 | 2 | 2 | 2 | 3 |
|  | THBS3 | THAP4 | THADA | TGFBI | TFEC | TEX10 | TET3 | TET2 | TEP1 | TENM4 |
| **patients  with mutations(n)** | 3 | 2 | 2 | 2 | 2 | 2 | 2 | 2 | 2 | 3 |
|  | TENM3 | TENM2 | TENM1 | TEKT4 | TEK | TECTA | TECPR2 | TCFL5 | TCF20 | TBX3 |
| **patients  with mutations(n)** | 2 | 2 | 2 | 2 | 2 | 2 | 2 | 2 | 2 | 3 |
|  | TBCD | TBC1D13 | TARBP1 | TAOK2 | TANGO6 | TANC2 | TAGAP | TAF6 | TAF1 | TACC2 |
| **patients  with mutations(n)** | 2 | 2 | 2 | 3 | 3 | 2 | 2 | 3 | 2 | 4 |
|  | TAB1 | TAAR5 | SZT2 | SYT12 | SYNPO2 | SYNM | SYNJ2 | SYNJ1 | SYNGAP1 | SYNE2 |
| **patients  with mutations(n)** | 2 | 2 | 4 | 2 | 2 | 2 | 2 | 3 | 2 | 2 |
|  | SYNE1 | SYNCRIP | SVIL | SV2B | SV2A | SUSD2 | SUPT5H | SUPT16H | SUN1 | STXBP5L |
| **patients  with mutations(n)** | 3 | 2 | 2 | 2 | 2 | 5 | 2 | 2 | 2 | 3 |
|  | STT3A | STRN | STRIP2 | STRC | STK40 | STK10 | STAT6 | STAG3 | STAB2 | STAB1 |
| **patients  with mutations(n)** | 2 | 2 | 2 | 2 | 2 | 2 | 3 | 2 | 3 | 4 |
|  | ST14 | SSRP1 | SSH1 | SRRM1 | SRFBP1 | SRF | SREBF2 | SRCAP | SPTBN4 | SPTBN1 |
| **patients  with mutations(n)** | 3 | 2 | 3 | 2 | 2 | 3 | 2 | 3 | 3 | 2 |
|  | SPTAN1 | SPHKAP | SPG7 | SPEN | SPATA5 | SPATA2 | SPATA18 | SPATA16 | SP1 | SOS1 |
| **patients  with mutations(n)** | 2 | 2 | 3 | 4 | 2 | 3 | 2 | 2 | 3 | 3 |
|  | SORL1 | SORCS3 | SORBS1 | SON | SOBP | SNX14 | SNW1 | SNRNP35 | SNRNP200 | SND1 |
| **patients  with mutations(n)** | 3 | 3 | 2 | 2 | 4 | 2 | 2 | 2 | 2 | 2 |
|  | SMPD3 | SMG6 | SMC1B | SMC1A | SMARCC2 | SMARCC1 | SMARCA4 | SMARCA2 | SLU7 | SLIT3 |
| **patients  with mutations(n)** | 2 | 3 | 2 | 2 | 2 | 3 | 3 | 2 | 3 | 4 |
|  | SLIT2 | SLC9B1 | SLC9A6 | SLC9A1 | SLC7A8 | SLC7A3 | SLC7A14 | SLC6A9 | SLC6A8 | SLC6A12 |
| **patients  with mutations(n)** | 2 | 3 | 2 | 2 | 2 | 2 | 2 | 2 | 2 | 2 |
|  | SLC5A9 | SLC5A4 | SLC4A3 | SLC4A2 | SLC4A11 | SLC45A4 | SLC40A1 | SLC39A5 | SLC39A4 | SLC38A5 |
| **patients  with mutations(n)** | 2 | 2 | 2 | 2 | 2 | 2 | 2 | 2 | 2 | 2 |
|  | SLC38A10 | SLC36A2 | SLC34A2 | SLC2A2 | SLC2A1 | SLC26A7 | SLC26A5 | SLC25A31 | SLC25A15 | SLC25A12 |
| **patients  with mutations(n)** | 2 | 2 | 2 | 2 | 2 | 2 | 2 | 2 | 2 | 2 |
|  | SLC25A10 | SLC24A3 | SLC24A1 | SLC22A7 | SLC22A23 | SLC22A2 | SLC17A8 | SLC17A7 | SLC13A3 | SLC13A2 |
| **patients  with mutations(n)** | 2 | 2 | 3 | 3 | 2 | 2 | 2 | 2 | 3 | 2 |
|  | SLC12A5 | SLC12A2 | SLC12A1 | SKIV2L | SIPA1L3 | SIPA1L2 | SIN3B | SIM2 | SIK3 | SIK2 |
| **patients  with mutations(n)** | 3 | 3 | 2 | 2 | 2 | 3 | 2 | 2 | 3 | 3 |

|  | SIDT2 | SHPRH | SHMT1 | SHISA6 | SHANK2 | SHANK1 | SH3RF1 | SH3PXD2B | SH3BP1 | SGSM2 |
| --- | --- | --- | --- | --- | --- | --- | --- | --- | --- | --- |
| **patients  with mutations(n)** | 2 | 3 | 2 | 2 | 2 | 2 | 2 | 2 | 2 | 2 |
|  | SGIP1 | SFSWAP | SF3B2 | SETX | SETD5 | SETD2 | SETD1B | SETBP1 | SESTD1 | SERPINB13 |
| **patients  with mutations(n)** | 2 | 3 | 3 | 2 | 3 | 2 | 2 | 3 | 2 | 2 |
|  | SERINC3 | SERINC2 | SEPT6 | SEPT3 | SEMA6A | SEMA5A | SEMA4G | SEMA4F | SEMA4C | SECISBP2L |
| **patients  with mutations(n)** | 2 | 3 | 2 | 2 | 2 | 2 | 2 | 2 | 2 | 2 |
|  | SEC61A2 | SEC31B | SEC24B | SEC23IP | SEC23B | SDK1 | SDHA | SDF2 | SDAD1 | SCYL1 |
| **patients  with mutations(n)** | 2 | 3 | 2 | 2 | 3 | 3 | 5 | 2 | 2 | 2 |
|  | SCNN1G | SCNN1B | SCN8A | SCN5A | SCN4A | SCN2A | SCN1A | SCN11A | SCN10A | SCARA5 |
| **patients  with mutations(n)** | 2 | 2 | 2 | 3 | 3 | 3 | 3 | 3 | 2 | 2 |
|  | SCAPER | SCAF4 | SBNO2 | SBF2 | SBF1 | SART3 | SAMD4B | SAFB | SACS | RYR3 |
| **patients  with mutations(n)** | 4 | 2 | 3 | 2 | 3 | 3 | 4 | 3 | 2 | 4 |
|  | RYR2 | RYR1 | RUSC2 | RUNX1T1 | RUNDC1 | RSF1 | RRP8 | RREB1 | RRAS | RPTOR |
| **patients  with mutations(n)** | 4 | 5 | 2 | 2 | 2 | 3 | 2 | 4 | 2 | 4 |
|  | RPS9 | RPS6KA5 | RPS6KA4 | RPS6KA2 | ROCK2 | ROCK1 | ROBO3 | ROBO1 | RNF5 | RNF220 |
| **patients  with mutations(n)** | 2 | 2 | 2 | 2 | 3 | 3 | 2 | 2 | 2 | 2 |
|  | RNF10 | RLF | RIN1 | RILPL1 | RIF1 | RICTOR | RHOBTB2 | RHBDF2 | RHBDF1 | RGL3 |
| **patients  with mutations(n)** | 3 | 2 | 2 | 2 | 2 | 2 | 2 | 2 | 2 | 2 |
|  | RFX5 | RFX4 | RFT1 | REV3L | RETSAT | RERE | REPS1 | RELN | RECQL | RC3H2 |
| **patients  with mutations(n)** | 2 | 2 | 2 | 2 | 2 | 5 | 2 | 4 | 2 | 3 |
|  | RBM33 | RBM28 | RBM26 | RBM20 | RBL2 | RBBP6 | RASSF7 | RASGRF2 | RASGRF1 | RASAL2 |
| **patients  with mutations(n)** | 3 | 3 | 2 | 2 | 3 | 3 | 2 | 2 | 3 | 2 |
|  | RASA1 | RAPGEF1 | RAP1GAP2 | RANBP3 | RANBP17 | RALGPS1 | RALGAPB | RALGAPA1 | RAI14 | RAI1 |
| **patients  with mutations(n)** | 2 | 2 | 2 | 2 | 3 | 2 | 2 | 3 | 2 | 2 |
|  | RAG1 | RAE1 | RAD54L2 | RAD54B | RABGAP1L | RABGAP1 | RABEP2 | R3HDM2 | QSER1 | QDPR |
| **patients  with mutations(n)** | 2 | 2 | 3 | 2 | 2 | 2 | 2 | 2 | 2 | 2 |
|  | PVRL2 | PUM1 | PTPRT | PTPRS | PTPRK | PTPRJ | PTPRG | PTPRF | PTPN3 | PTPN23 |
| **patients  with mutations(n)** | 2 | 2 | 2 | 2 | 2 | 2 | 3 | 2 | 2 | 2 |
|  | PTPN14 | PTPDC1 | PTGS2 | PTDSS1 | PTCD3 | PSTPIP1 | PSME4 | PSMD9 | PSD | PRSS16 |
| **patients  with mutations(n)** | 3 | 2 | 2 | 2 | 2 | 2 | 2 | 2 | 2 | 2 |
|  | PRR15L | PRR14 | PRPH | PRPF8 | PRPF40A | PRMT5 | PRL | PRKG2 | PRKD2 | PRKAA2 |
| **patients  with mutations(n)** | 2 | 3 | 2 | 4 | 2 | 2 | 2 | 2 | 2 | 2 |
|  | PREX2 | PREX1 | PRDM15 | PRDM10 | PPTC7 | PPP1R9A | PPOX | PPM1L | PPM1J | PPIP5K2 |

| **patients  with mutations(n)** | 2 | 4 | 2 | 4 | 2 | 2 | 2 | 2 | 2 | 2 |
| --- | --- | --- | --- | --- | --- | --- | --- | --- | --- | --- |
|  | PPIL2 | PPFIA4 | PPARGC1B | POU2F1 | POLR3B | POLR3A | POLR1B | POLG | POLE | POLDIP3 |
| **patients  with mutations(n)** | 2 | 2 | 4 | 2 | 2 | 2 | 2 | 2 | 5 | 2 |
|  | POLD1 | POLA1 | PODN | POC1A | PNPLA6 | PMPCB | PMEL | PLXND1 | PLXNC1 | PLXNB1 |
| **patients  with mutations(n)** | 2 | 3 | 2 | 2 | 3 | 2 | 2 | 2 | 2 | 2 |
|  | PLXNA4 | PLXNA3 | PLXNA2 | PLXDC2 | PLS1 | PLOD3 | PLIN5 | PLEKHH2 | PLEKHG3 | PLEKHA6 |
| **patients  with mutations(n)** | 2 | 3 | 2 | 2 | 2 | 3 | 2 | 2 | 2 | 2 |
|  | PLEKHA4 | PLEKHA1 | PLEC | PLD1 | PLCH1 | PLCE1 | PLCB4 | PLCB3 | PLCB1 | PLB1 |
| **patients  with mutations(n)** | 2 | 2 | 4 | 3 | 4 | 2 | 2 | 3 | 2 | 4 |
|  | PLA2G4D | PKP4 | PKNOX2 | PKN3 | PKD1 | PITRM1 | PITPNM3 | PITPNM2 | PITPNM1 | PIR |
| **patients  with mutations(n)** | 3 | 3 | 2 | 2 | 2 | 2 | 2 | 4 | 3 | 2 |
|  | PIKFYVE | PIK3R5 | PIK3CD | PIGZ | PIGT | PIGA | PIEZO2 | PHLPP2 | PHLPP1 | PHLDB1 |
| **patients  with mutations(n)** | 2 | 2 | 2 | 2 | 2 | 2 | 2 | 2 | 2 | 3 |
|  | PHKG1 | PHKA1 | PHIP | PHF21B | PHF14 | PHC3 | PHC2 | PHC1 | PGM5 | PFKP |
| **patients  with mutations(n)** | 2 | 2 | 3 | 3 | 2 | 3 | 2 | 3 | 2 | 2 |
|  | PFKFB4 | PFKFB3 | PFAS | PES1 | PDZRN3 | PDXP | PDPR | PDK2 | PDIA6 | PDE4A |
| **patients  with mutations(n)** | 2 | 3 | 5 | 2 | 2 | 2 | 2 | 2 | 2 | 2 |
|  | PDE1C | PDE1B | PDCD11 | PCYT1A | PCSK7 | PCNXL4 | PCM1 | PCLO | PCED1A | PCDHB13 |
| **patients  with mutations(n)** | 2 | 2 | 3 | 2 | 2 | 2 | 2 | 2 | 2 | 2 |
|  | PCDHA9 | PCDH12 | PCBP4 | PC | PBRM1 | PAXIP1 | PAX7 | PARS2 | PARP14 | PARD3 |
| **patients  with mutations(n)** | 2 | 2 | 3 | 3 | 3 | 2 | 2 | 2 | 4 | 2 |
|  | PAQR7 | PAPPA | PAN3 | PAK6 | P4HA2 | OVGP1 | OTOF | OSBPL11 | OS9 | OR2D3 |
| **patients  with mutations(n)** | 2 | 2 | 3 | 2 | 2 | 2 | 3 | 2 | 2 | 2 |
|  | OPRM1 | OPRL1 | OPHN1 | OLFML2A | OGDH | ODF1 | OBSCN | NYAP2 | NYAP1 | NXF1 |
| **patients  with mutations(n)** | 2 | 2 | 2 | 4 | 2 | 2 | 3 | 3 | 2 | 2 |
|  | NUP98 | NUP43 | NUP214 | NUP205 | NUP188 | NUDT22 | NUDT14 | NUAK1 | NTRK3 | NTRK1 |
| **patients  with mutations(n)** | 2 | 2 | 2 | 2 | 3 | 2 | 2 | 2 | 2 | 2 |
|  | NTAN1 | NRXN3 | NRXN2 | NRXN1 | NRP2 | NRP1 | NRDE2 | NRCAM | NRAP | NPR3 |
| **patients  with mutations(n)** | 2 | 2 | 2 | 3 | 2 | 2 | 2 | 2 | 2 | 2 |
|  | NPNT | NPHP3 | NPBWR1 | NONO | NOL6 | NMUR2 | NME7 | NLGN1 | NID2 | NHS |
| **patients  with mutations(n)** | 4 | 2 | 3 | 2 | 3 | 3 | 2 | 2 | 2 | 2 |
|  | NFIX | NFIB | NFIA | NFATC4 | NFATC2 | NFATC1 | NFAT5 | NFASC | NEUROD2 | NEURL4 |
| **patients  with mutations(n)** | 3 | 2 | 3 | 2 | 3 | 2 | 2 | 3 | 2 | 2 |
|  | NEIL3 | NEDD4 | NEB | NDUFV1 | NDUFB7 | NDUFA7 | NDST1 | NDRG4 | NDRG1 | NCOR1 |
| **patients  with mutations(n)** | 2 | 2 | 3 | 2 | 2 | 3 | 3 | 2 | 4 | 4 |
|  | NCOA7 | NCOA6 | NCKAP1L | NCF4 | NCAM2 | NBR1 | NBAS | NAV3 | NAV2 | NAV1 |
| **patients  with mutations(n)** | 2 | 2 | 2 | 2 | 3 | 2 | 5 | 2 | 3 | 3 |
|  | NADSYN1 | NAA15 | MYOT | MYOM2 | MYOM1 | MYOF | MYOCD | MYO5C | MYO5B | MYO5A |
| **patients  with mutations(n)** | 2 | 2 | 2 | 2 | 2 | 3 | 2 | 3 | 3 | 2 |
|  | MYO1F | MYO1A | MYO18B | MYO18A | MYO10 | MYLK2 | MYLK | MYH8 | MYH7 | MYH6 |
| **patients  with mutations(n)** | 2 | 2 | 4 | 3 | 3 | 2 | 2 | 2 | 3 | 3 |
|  | MYH4 | MYH2 | MYH14 | MYH13 | MYH11 | MYH10 | MYCN | MYCBP2 | MYBPC3 | MYBPC1 |
| **patients  with mutations(n)** | 4 | 2 | 6 | 3 | 7 | 3 | 2 | 4 | 5 | 2 |
|  | MUT | MUSK | MTSS1 | MTMR2 | MTMR11 | MTG2 | MSN | MRPS18B | MROH7 | MPRIP |
| **patients  with mutations(n)** | 2 | 2 | 3 | 2 | 2 | 2 | 2 | 2 | 3 | 2 |
|  | MPPED1 | MPP2 | MMS22L | MMP27 | MLXIPL | MLXIP | MLH1 | MIPEP | MINK1 | MIDN |
| **patients  with mutations(n)** | 2 | 2 | 2 | 2 | 2 | 2 | 2 | 2 | 2 | 2 |
|  | MICALL1 | MICAL3 | MIB2 | MIA3 | MGA | MFSD3 | MFGE8 | MEX3B | MEIS3 | MEGF8 |
| **patients  with mutations(n)** | 2 | 2 | 3 | 2 | 3 | 2 | 3 | 2 | 2 | 6 |
|  | MEGF6 | MEGF11 | MED6 | MED23 | MED13 | MED12L | MED12 | MECOM | MDN1 | MDC1 |
| **patients  with mutations(n)** | 3 | 2 | 2 | 2 | 3 | 4 | 3 | 3 | 3 | 3 |
|  | MCRS1 | MCM8 | MCM5 | MCM3AP | MCM2 | MBTPS2 | MBTD1 | MBD6 | MATN4 | MAST4 |
| **patients  with mutations(n)** | 2 | 3 | 2 | 2 | 2 | 2 | 2 | 2 | 2 | 2 |
|  | MAST3 | MAST2 | MAST1 | MASP1 | MARK3 | MARK2 | MAPT | MAPKBP1 | MAPK7 | MAP7D1 |
| **patients  with mutations(n)** | 5 | 2 | 3 | 4 | 2 | 2 | 4 | 2 | 2 | 2 |
|  | MAP4K1 | MAP3K9 | MAP3K6 | MAP3K10 | MAP1B | MAP1A | MAN2B1 | MAN2A1 | MAN1C1 | MAGI3 |
| **patients  with mutations(n)** | 2 | 2 | 2 | 3 | 2 | 2 | 3 | 3 | 3 | 3 |
|  | MADD | MACF1 | LYST | LSM14B | LRRN2 | LRRN1 | LRRC4B | LRP6 | LRP5 | LRP4 |
| **patients  with mutations(n)** | 3 | 2 | 2 | 3 | 2 | 2 | 3 | 2 | 3 | 2 |
|  | LRP2 | LRP1B | LRP1 | LRFN4 | LRBA | LPIN3 | LPHN2 | LPHN1 | LPAR3 | LOXL4 |
| **patients  with mutations(n)** | 2 | 4 | 6 | 4 | 4 | 2 | 2 | 2 | 2 | 3 |
|  | LOXHD1 | LONP1 | LMTK2 | LMOD3 | LMO7 | LMBRD1 | LIPG | LINGO1 | LIG3 | LGI2 |
| **patients  with mutations(n)** | 2 | 3 | 4 | 3 | 2 | 2 | 2 | 2 | 2 | 2 |
|  | LETM1 | LENG8 | LDLR | LDB3 | LBX1 | LATS1 | LAMB2 | LAMB1 | LAMA3 | LAMA2 |
| **patients  with mutations(n)** | 2 | 2 | 3 | 3 | 2 | 2 | 3 | 2 | 2 | 4 |
|  | LAMA1 | L3MBTL2 | KTN1 | KRT75 | KRT6B | KRT35 | KRT24 | KRT19 | KRT12 | KRIT1 |

| **patients  with mutations(n)** | 4 | 2 | 2 | 2 | 3 | 2 | 3 | 2 | 2 | 2 |
| --- | --- | --- | --- | --- | --- | --- | --- | --- | --- | --- |
|  | KPTN | KPNA7 | KMT2E | KMT2D | KMT2C | KMT2B | KMT2A | KLHL40 | KLHL3 | KLHL17 |
| **patients  with mutations(n)** | 2 | 2 | 2 | 2 | 10 | 4 | 5 | 2 | 2 | 3 |
|  | KLHL12 | KLHDC1 | KLC4 | KIF7 | KIF5C | KIF5B | KIF5A | KIF4A | KIF26B | KIF21A |
| **patients  with mutations(n)** | 2 | 2 | 2 | 3 | 3 | 2 | 2 | 2 | 2 | 2 |
|  | KIF20B | KIF1C | KIF1B | KIF13A | KIDINS220 | KIAA2018 | KIAA1468 | KIAA1239 | KIAA1147 | KIAA1109 |
| **patients  with mutations(n)** | 2 | 3 | 2 | 3 | 2 | 3 | 2 | 2 | 2 | 3 |
|  | KIAA1024 | KIAA0947 | KIAA0895 | KIAA0586 | KIAA0430 | KIAA0226 | KIAA0100 | KEL | KDM6B | KDM6A |
| **patients  with mutations(n)** | 2 | 2 | 2 | 3 | 2 | 2 | 3 | 3 | 3 | 2 |
|  | KDM5A | KDM2A | KCTD3 | KCNK5 | KCNJ5 | KCNJ18,  KCNJ12 | KCNJ12,  KCNJ18 | KCNH5 | KCNH1 | KCNG1 |
| **patients  with mutations(n)** | 2 | 2 | 2 | 2 | 3 | 3 | 3 | 2 | 2 | 2 |
|  | KCMF1 | KAT8 | KAT6B | KANK4 | KANK1 | KALRN | JPH2 | JMJD1C | JARID2 | JAKMIP2 |
| **patients  with mutations(n)** | 2 | 2 | 3 | 2 | 2 | 4 | 3 | 4 | 3 | 3 |
|  | JAKMIP1 | ITSN2 | ITSN1 | ITPR1 | ITGB5 | ITGB2 | ITGA7 | ITGA6 | ITGA2B | ITGA2 |
| **patients  with mutations(n)** | 2 | 2 | 2 | 6 | 2 | 2 | 2 | 2 | 2 | 2 |
|  | IQSEC1 | IQGAP3 | IQGAP2 | IQGAP1 | IPO8 | IP6K2 | INTS7 | INTS1 | INSRR | INPP5J |
| **patients  with mutations(n)** | 2 | 5 | 3 | 2 | 3 | 2 | 2 | 2 | 2 | 5 |
|  | INPP5B | IMPG2 | IMMT | IKBIP | IGDCC4 | IFT172 | IFRD1 | IBTK | IARS | HUWE1 |
| **patients  with mutations(n)** | 3 | 2 | 2 | 2 | 2 | 3 | 2 | 2 | 2 | 3 |
|  | HSPG2 | HSPA9 | HSPA1L | HSP90AB1 | HSP90AA1 | HSD11B2 | HS3ST6 | HPS5 | HPS4 | HPS3 |
| **patients  with mutations(n)** | 2 | 2 | 2 | 2 | 2 | 2 | 2 | 2 | 2 | 3 |
|  | HNRNPUL2 | HNRNPUL1 | HNRNPU | HNF4A | HMGXB3 | HMGCR | HMCN1 | HK1 | HIRA | HIPK4 |
| **patients  with mutations(n)** | 2 | 2 | 3 | 2 | 3 | 4 | 2 | 2 | 4 | 2 |
|  | HIPK2 | HIPK1 | HHATL | HEXA | HERC3 | HERC2 | HERC1 | HECTD1 | HEATR2 | HEATR1 |
| **patients  with mutations(n)** | 2 | 2 | 2 | 2 | 2 | 3 | 4 | 2 | 2 | 2 |
|  | HDHD2 | HDAC9 | HCLS1 | HCFC1 | GZMA | GUSB | GUCY2F | GTPBP2 | GTPBP1 | GTF2I |
| **patients  with mutations(n)** | 2 | 2 | 3 | 3 | 2 | 2 | 2 | 2 | 2 | 2 |
|  | GSTO1 | GSE1 | GRSF1 | GRN | GRM1 | GRK6 | GRIN3B | GRIK4 | GRIA4 | GRIA2 |
| **patients  with mutations(n)** | 2 | 5 | 2 | 2 | 3 | 2 | 2 | 2 | 2 | 2 |
|  | GRHL3 | GRHL2 | GREB1L | GPRC5A | GPR98 | GPR61 | GPR45 | GPR37L1 | GPR126 | GPR124 |
| **patients  with mutations(n)** | 2 | 2 | 3 | 2 | 5 | 2 | 2 | 2 | 2 | 2 |
|  | GPR115 | GPR114 | GPHN | GPC3 | GPBP1 | GON4L | GOLGA3 | GNB3 | GNAO1 | GNAI2 |
| **patients  with mutations(n)** | 2 | 2 | 3 | 2 | 2 | 2 | 3 | 3 | 2 | 3 |
|  | GLTSCR1L | GLS2 | GLI1 | GLE1 | GJB5 | GJA5 | GIPC1 | GIGYF2 | GFM2 | GFM1 |
| **patients  with mutations(n)** | 3 | 2 | 3 | 2 | 2 | 2 | 2 | 3 | 2 | 2 |
|  | GFI1B | GCN1L1 | GCFC2 | GBGT1 | GBF1 | GATAD2A | GAREM | GAPVD1 | GAPDH | GALNT3 |
| **patients  with mutations(n)** | 2 | 3 | 2 | 3 | 2 | 2 | 2 | 2 | 2 | 2 |
|  | GALC | GABRR1 | GABBR2 | FZD9 | FYCO1 | FXR2 | FURIN | FUCA2 | FTSJ3 | FRYL |
| **patients  with mutations(n)** | 2 | 3 | 2 | 2 | 3 | 4 | 2 | 2 | 3 | 3 |
|  | FRMPD4 | FREM1 | FOXP2 | FOXP1 | FOSB | FOS | FNDC1 | FN1 | FLNB | FLII |
| **patients  with mutations(n)** | 2 | 2 | 2 | 2 | 2 | 2 | 2 | 3 | 4 | 3 |
|  | FKBP15 | FIP1L1 | FILIP1L | FILIP1 | FHOD3 | FHOD1 | FGFR4 | FGD5 | FGD3 | FER |
| **patients  with mutations(n)** | 2 | 2 | 2 | 3 | 3 | 2 | 2 | 3 | 2 | 2 |
|  | FEM1A | FBXW8 | FBXO31 | FBXO10 | FBXL6 | FBXL19 | FBN2 | FBN1 | FBLN1 | FAT4 |
| **patients  with mutations(n)** | 2 | 2 | 2 | 3 | 2 | 2 | 3 | 2 | 2 | 5 |
|  | FAT3 | FAT2 | FASN | FARP1 | FANCI | FAM73B | FAM65B | FAM65A | FAM3A | FAM169A |
| **patients  with mutations(n)** | 2 | 3 | 2 | 3 | 2 | 4 | 2 | 2 | 2 | 2 |
|  | FAM149B1 | FAM13B | F8 | EXPH5 | EXOC6B | EXOC2 | EVI5L | EVI5 | ETV5 | ESRP2 |
| **patients  with mutations(n)** | 2 | 3 | 2 | 2 | 2 | 3 | 3 | 2 | 2 | 2 |
|  | ESRP1 | ERI3 | ERGIC1 | ERCC2 | ERBB4 | ERBB2IP | EPX | EPRS | EPN1 | EPHB3 |
| **patients  with mutations(n)** | 2 | 2 | 2 | 3 | 2 | 3 | 2 | 4 | 2 | 4 |
|  | EPHA7 | EPG5 | EPB41L4B | EP400 | EP300 | ENPP2 | ENOX2 | ENO2 | ENKUR | EML3 |
| **patients  with mutations(n)** | 2 | 2 | 2 | 2 | 3 | 2 | 3 | 3 | 2 | 2 |
|  | EML2 | EMC1 | ELMO2 | ELMO1 | ELL | ELFN1 | EIF5B | EIF3A | EHMT1 | EGFLAM |
| **patients  with mutations(n)** | 2 | 3 | 2 | 2 | 2 | 2 | 2 | 3 | 3 | 2 |
|  | EEF2K | EEF1D | E2F8 | DYSF | DYRK1A | DYNC2H1 | DYNC1H1 | DUOX1 | DTX2 | DTNB |
| **patients  with mutations(n)** | 3 | 2 | 2 | 6 | 3 | 3 | 5 | 2 | 2 | 3 |
|  | DSCAM | DRP2 | DRGX | DPYSL5 | DPYSL2 | DPP10 | DPH1 | DPEP2 | DOLPP1 | DOK1 |
| **patients  with mutations(n)** | 2 | 2 | 2 | 2 | 4 | 2 | 2 | 2 | 2 | 2 |
|  | DOCK8 | DOCK7 | DOCK3 | DOCK10 | DOCK1 | DOC2A | DNMT3B | DNMBP | DNM3 | DNM2 |
| **patients  with mutations(n)** | 4 | 3 | 3 | 3 | 2 | 3 | 2 | 2 | 2 | 4 |
|  | DNM1 | DNER | DNAJC1 | DNAJB9 | DNAJB13 | DNAH9 | DNAH8 | DNAH7 | DNAH6 | DNAH5 |
| **patients  with mutations(n)** | 3 | 2 | 2 | 2 | 2 | 3 | 4 | 3 | 3 | 3 |
|  | DNAH3 | DNAH2 | DNAH12 | DNAH11 | DNAH10 | DNAH1 | DMXL2 | DMWD | DMRT1 | DLL1 |
| **patients  with mutations(n)** | 5 | 3 | 3 | 3 | 6 | 3 | 2 | 2 | 2 | 2 |
|  | DLK2 | DLGAP4 | DLGAP1 | DLG5 | DLG4 | DLG1 | DLC1 | DIP2B | DIP2A | DIDO1 |
| **patients  with mutations(n)** | 3 | 3 | 4 | 2 | 2 | 2 | 2 | 2 | 2 | 3 |
|  | DICER1 | DIAPH3 | DIAPH2 | DHX38 | DHX37 | DHX34 | DHX16 | DHTKD1 | DHRS1 | DHODH |
| **patients  with mutations(n)** | 2 | 2 | 2 | 2 | 2 | 3 | 3 | 2 | 2 | 3 |
|  | DHDDS | DHCR24 | DGKD | DEPDC5 | DENND5A | DENND4B | DENND4A | DENND3 | DENND1A | DEF6 |
| **patients  with mutations(n)** | 2 | 2 | 3 | 2 | 2 | 2 | 2 | 2 | 2 | 2 |
|  | DDX47 | DDX27 | DDX19A | DDX10 | DCHS1 | DCBLD1 | DCAF8 | DCAF5 | DBX2 | DAW1 |
| **patients  with mutations(n)** | 2 | 2 | 3 | 2 | 4 | 2 | 2 | 2 | 2 | 2 |
|  | DAGLA | DAG1 | CYTH2 | CYP2S1 | CYP11A1 | CYB5R2 | CWF19L2 | CUX2 | CUX1 | CUL9 |
| **patients  with mutations(n)** | 3 | 3 | 2 | 2 | 2 | 2 | 2 | 3 | 3 | 3 |
|  | CUL7 | CUL4B | CUBN | CTSH | CTNND2 | CTNNA3 | CTIF | CSTF2 | CSRNP1 | CSPP1 |
| **patients  with mutations(n)** | 6 | 3 | 3 | 2 | 3 | 2 | 2 | 2 | 2 | 3 |
|  | CSMD1 | CROT | CREBBP | CPOX | CPB2 | CORIN | COPG1 | COPA | COL7A1 | COL6A6 |
| **patients  with mutations(n)** | 3 | 2 | 2 | 2 | 2 | 2 | 2 | 4 | 2 | 2 |
|  | COL6A3 | COL5A2 | COL4A5 | COL4A4 | COL4A3 | COL4A2 | COL28A1 | COL27A1 | COL16A1 | COL12A1 |
| **patients  with mutations(n)** | 3 | 2 | 2 | 2 | 2 | 3 | 2 | 3 | 3 | 2 |
|  | COL11A2 | COL11A1 | CNTNAP4 | CNTNAP1 | CNTN2 | CNR1 | CNOT6 | CNOT3 | CNGB1 | CNGA1 |
| **patients  with mutations(n)** | 3 | 3 | 2 | 2 | 3 | 2 | 2 | 2 | 5 | 2 |
|  | CMKLR1 | CLUH | CLTCL1 | CLPTM1 | CLPB | CLIP4 | CLIP2 | CLEC16A | CLDN10 | CLCNKA |
| **patients  with mutations(n)** | 2 | 2 | 3 | 2 | 3 | 2 | 3 | 3 | 2 | 2 |
|  | CLCN2 | CLCA2 | CLCA1 | CLASRP | CKAP5 | CILP | CIART | CHRNA7 | CHRNA1 | CHPF |
| **patients  with mutations(n)** | 2 | 2 | 2 | 2 | 3 | 2 | 2 | 2 | 2 | 2 |
|  | CHMP5 | CHFR | CHD9 | CHD8 | CHD7 | CHD6 | CHD4 | CHD3 | CES1 | CEP350 |
| **patients  with mutations(n)** | 2 | 2 | 4 | 4 | 2 | 2 | 4 | 2 | 2 | 2 |
|  | CEP290 | CELSR3 | CELSR1 | CELF5 | CELF2 | CELF1 | CEBPZ | CDK12 | CDH5 | CDH4 |
| **patients  with mutations(n)** | 3 | 3 | 4 | 2 | 2 | 2 | 2 | 2 | 2 | 3 |
|  | CDH24 | CDH23 | CDH1 | CDCA7L | CDC7 | CDC42BPG | CDC42BPB | CDC42BPA | CDC23 | CD200R1 |
| **patients  with mutations(n)** | 2 | 2 | 2 | 2 | 2 | 2 | 3 | 2 | 2 | 2 |
|  | CCSER2 | CCNJL | CCDC88C | CCDC88A | CCDC85A | CCDC63 | CCDC141 | CCDC106 | CCDC102A | CC2D1A |
| **patients  with mutations(n)** | 2 | 2 | 2 | 3 | 2 | 2 | 2 | 2 | 2 | 2 |
|  | CBY1 | CBLB | CBL | CASR | CASKIN2 | CARHSP1 | CAP2 | CAMTA1 | CAMSAP3 | CAMSAP1 |
| **patients  with mutations(n)** | 2 | 3 | 2 | 2 | 3 | 2 | 2 | 3 | 2 | 2 |
|  | CAMKV | CAMK1 | CALR3 | CAD | CACNG1 | CACNB3 | CACNB2 | CACNB1 | CACNA2D3 | CACNA2D1 |
| **patients  with mutations(n)** | 2 | 2 | 2 | 3 | 2 | 2 | 2 | 2 | 3 | 2 |
|  | CACNA1I | CACNA1G | CACNA1F | CACNA1E | CACNA1D | CACNA1C | CACNA1B | CACNA1A | CABP7 | CA11 |
| **patients  with mutations(n)** | 2 | 3 | 4 | 4 | 3 | 4 | 3 | 3 | 2 | 2 |
|  | C3orf67 | C2CD5 | C20orf26 | C1orf173 | C17orf85 | C17orf64 | C10orf2 | C10orf118 | BTBD11 | BSPRY |
| **patients  with mutations(n)** | 2 | 3 | 2 | 2 | 2 | 2 | 2 | 3 | 2 | 2 |
|  | BSN | BSDC1 | BRWD1 | BRPF1 | BRCA1 | BNC1 | BMS1 | BMPER | BIVM-ERCC5,ERCC5 | BICD1 |
| **patients  with mutations(n)** | 3 | 2 | 2 | 2 | 2 | 2 | 2 | 2 | 2 | 3 |
|  | BICC1 | BFSP1 | BEND4 | BCORL1 | BCOR | BCL9 | BCL6 | BCKDHA | BCCIP | BCAS3 |
| **patients  with mutations(n)** | 2 | 2 | 2 | 3 | 2 | 2 | 2 | 2 | 2 | 2 |
|  | BCAS1 | BCAN | BAZ2A | BAI2 | B4GALT5 | B3GALTL | AZI1 | AURKAIP1 | ATXN7L1 | ATXN2L |
| **patients  with mutations(n)** | 2 | 2 | 2 | 3 | 2 | 2 | 2 | 2 | 2 | 5 |
|  | ATRNL1 | ATRN | ATRIP | ATP9B | ATP9A | ATP8B4 | ATP8A2 | ATP5A1 | ATP2B3 | ATP1A4 |
| **patients  with mutations(n)** | 2 | 3 | 2 | 3 | 4 | 2 | 2 | 2 | 2 | 3 |
|  | ATP1A3 | ATP1A2 | ATP10A | ATN1 | ATM | ATG4B | ATG2A | ATF7IP | ATF6 | ATAD3A |
| **patients  with mutations(n)** | 2 | 2 | 2 | 2 | 2 | 2 | 3 | 2 | 2 | 2 |
|  | ATAD2 | ASXL1 | ASTN1 | ASPM | ASPDH | ASIC1 | ASH1L | ASCC3 | ASB12 | ASAP2 |
| **patients  with mutations(n)** | 2 | 2 | 6 | 2 | 2 | 2 | 7 | 2 | 2 | 3 |
|  | ARSF | ARPP21 | ARPC2 | ARNTL | ARMC3 | ARID4B | ARID1B | ARID1A | ARHGEF5 | ARHGEF40 |
| **patients  with mutations(n)** | 2 | 2 | 2 | 2 | 2 | 4 | 2 | 2 | 3 | 3 |
|  | ARHGEF18 | ARHGEF17 | ARHGEF12 | ARHGAP9 | ARHGAP42 | ARHGAP35 | ARHGAP20 | ARAP3 | ARAP2 | ARAF |
| **patients  with mutations(n)** | 3 | 2 | 3 | 2 | 2 | 2 | 2 | 2 | 2 | 2 |
|  | APP | APOM | APOB | APOA5 | APLF | APCDD1 | APC2 | APC | APBB2 | APBA2 |
| **patients  with mutations(n)** | 2 | 2 | 3 | 2 | 3 | 2 | 2 | 2 | 2 | 2 |
|  | APBA1 | APAF1 | AP4M1 | AP3B2 | AP2A2 | AP2A1 | AP1B1 | ANXA9 | ANXA4 | ANO8 |
| **patients  with mutations(n)** | 2 | 3 | 2 | 2 | 2 | 2 | 2 | 2 | 2 | 2 |
|  | ANO5 | ANLN | ANKS1A | ANKRD28 | ANKRD17 | ANKRD12 | ANKRD11 | ANK3 | ANK2 | ANAPC5 |
| **patients  with mutations(n)** | 3 | 2 | 4 | 2 | 3 | 2 | 4 | 4 | 4 | 2 |
|  | ANAPC4 | ANAPC2 | AMOTL2 | ALX3 | ALS2 | ALPK1 | ALOX12B | ALK | ALDOA | ALDH8A1 |

| **patients  with mutations(n)** | 2 | 3 | 2 | 2 | 3 | 3 | 2 | 2 | 2 | 2 |
| --- | --- | --- | --- | --- | --- | --- | --- | --- | --- | --- |
|  | ALDH2 | ALDH1L1 | ALDH18A1 | AKAP8 | AKAP11 | AK9 | AJAP1 | AIRE | AIDA | AHNAK2 |
| **patients  with mutations(n)** | 2 | 2 | 3 | 2 | 2 | 2 | 2 | 2 | 2 | 2 |
|  | AHCYL1 | AGO2 | AGO1 | AGFG2 | AGBL1 | AGAP2 | AFF4 | AFF3 | AFAP1L1 | AFAP1 |
| **patients  with mutations(n)** | 2 | 2 | 4 | 2 | 3 | 3 | 3 | 2 | 3 | 2 |
|  | ADPRHL1 | ADCYAP1R1 | ADCY9 | ADCY6 | ADCY3 | ADCY2 | ADCY1 | ADAMTSL3 | ADAMTS9 | ADAMTS3 |
| **patients  with mutations(n)** | 2 | 2 | 2 | 4 | 3 | 2 | 3 | 4 | 4 | 2 |
|  | ADAMTS2 | ACTN4 | ACTN2 | ACSM5 | ACSL6 | ACSL4 | ACOT12 | ACOT11 | ACIN1 | ACE |
| **patients  with mutations(n)** | 2 | 2 | 2 | 2 | 2 | 3 | 2 | 2 | 3 | 2 |
|  | ACAP2 | ACADSB | ACACB | ABTB2 | ABR | ABLIM3 | ABLIM1 | ABHD16A | ABCD1 | ABCC8 |
| **patients  with mutations(n)** | 2 | 2 | 2 | 2 | 2 | 2 | 2 | 2 | 2 | 2 |
|  | ABCC5 | ABCC11 | ABCC1 | ABCB8 | ABCB6 | ABCB11 | ABCB10 | ABCB1 | ABCA8 | ABCA7 |
| **patients  with mutations(n)** | 3 | 3 | 4 | 3 | 3 | 2 | 2 | 2 | 2 | 2 |
|  | ABCA4 | ABCA2 | ABCA13 | ABCA12 | ABCA1 | ABAT | AATK | AAK1 | A1CF |  |
| **patients  with mutations(n)** | 4 | 3 | 2 | 2 | 3 | 2 | 2 | 2 | 2 |  |

**Table F3** Summary of Likely Pathogenic mutations based on ACMG guidelines

| Gene | SNV/Indel | REF | ALT | Region | Transcript ID | Exonic Function | Nucleotide change | Chr: Position | Pathogenicity |
| --- | --- | --- | --- | --- | --- | --- | --- | --- | --- |
| ABCA4 | rs201855602 | G | A | exonic | NM_000350 | missense SNV | exon21:c.C3056T | 1p22.1 | Pathogenic |
| ABCB11 | rs72549398 | G | A | exonic | NM_003742 | missense SNV | exon24:c.C3148T | 2q31.1 | Pathogenic |
| ABCD1 | rs886044777 | G | A | exonic | NM_000033 | missense SNV | exon3:c.G1166A | Xq28 | Pathogenic |
| ACAD9 | rs368949613 | C | T | exonic | NM_014049 | missense SNV | exon12:c.C1249T | 3q21.3 | Pathogenic |
| AR | rs137852562 | C | T | exonic | NM_000044, NM_001011645 | missense SNV | exon6:c.C2323T, exon6:c.C727T | Xq12 | Pathogenic |
| BIVM-ERCC5, ERCC5 |  | C | T | exonic | NM_000123, NM_001204425 | missense SNV | exon6:c.C640T, exon14:c.C2002T | 13q33.1 | Likely pathogenic |
| BIVM-ERCC5, ERCC5 |  | A | G | exonic |  | missense SNV | exon7:c.A767G, exon15:c.A2129G | 13q33.1 | Likely pathogenic |
| CASR | rs193922423 | G | A | exonic | NM_000388, NM_001178065 | missense SNV | exon5:c.G1525A | 3q21.1 | Pathogenic; |
| CHRNA4 | rs121912243 | G | A | exonic | NM_000744 | missense SNV | exon5:c.C442T | 20q13.33 | Likely pathogenic |
| COL4A5 | rs104886237 | G | A | exonic | NM_000495, NM_033380 | missense SNV | exon39:c.G3508A | Xq22.3 | Pathogenic |
| COQ2 | rs761785906 | G | A | exonic | NM_015697 | missense SNV | exon6:c.C950T | 4q21.23 | Pathogenic |
| EDA | rs142948132 | G | A | exonic | NM_001005609, NM_001005612, NM_001399 | missense SNV | exon8:c.G995A, exon8:c.G986A, exon8:c.G1001A | Xq13.1 | Pathogenic |
| EGFR | rs121913465 | G | T | exonic | NM_005228 | missense SNV | exon20:c.G2303T | 7p11.2 | Likely pathogenic |
| EIF2B3 | rs141988913 | C | T | exonic | NM_001166588, NM_001261418, NM_020365 | missense SNV | exon3:c.G272A | 1p34.1 | Likely pathogenic |
| ERCC2 | rs121913026 | G | A | exonic | NM_000400 | missense SNV | exon22:c.C2164T | 19q13.32 | Pathogenic |
| FBN2 | rs368384428 | C | T | exonic | NM_001999 | missense SNV | exon45:c.G5678A | 5q23.3 | Likely pathogenic |
| FBN2 | rs772994944 | C | T | exonic |  | missense SNV | exon48:c.G6073A | 5q23.3 | Likely pathogenic |
| FLG | rs578184315 | CATGGGATG | C | exonic | NM_002016 | frameshift deletion | exon3:c.6950_6957del | 1q21.3 | Pathogenic; |
| GJA3 | . | G | A | exonic | NM_021954 | missense SNV | exon2:c.C56T | 13q12.11 | Pathogenic |
| GUSB | rs121918174 | G | A | exonic | NM_000181 | missense SNV | exon4:c.C646T | 7q11.21 | Pathogenic |
| HOGA1 | rs150702945 | G | A | exonic | NM_138413 | missense SNV | exon2:c.G337A | 10q24.2 | Pathogenic; |
| INSR | rs121913156 | C | T | exonic | NM_000208, NM_001079817 | missense SNV | exon19:c.G3566A, exon20:c.G3602A | 19p13.2 | Pathogenic |
| JAG1 | rs121918350 | G | A | exonic | NM_000214 | missense SNV | exon4:c.C550T | 20p12.2 | Pathogenic |
| KMT2C | rs150073007 | G | GT | exonic | NM_170606 | stopgain | exon14:c.2447dupA | 7q36.1 | Likely pathogenic |
| KRT14 | rs58330629 | C | T | exonic | NM_000526 | missense SNV | exon1:c.G374A | 17q21.2 | Pathogenic; |
| LBR | rs200180113 | G | A | exonic | NM_002296, NM_194442 | missense SNV | exon9:c.C1114T | 1q42.12 | Pathogenic |
| LDB3 | rs121908335 | C | T | exonic | NM_001080115, NM_001080116, NM_001171611 | missense SNV | exon7:c.C943T, exon8:c.C802T, exon8:c.C1147T | 10q23.2 | Pathogenic |
| LDLR | rs746834464 | G | A | exonic | NM_000527, NM_001195798, NM_001195799, NM_001195800, NM_001195803 | missense SNV | exon5:c.G445A, exon6:c.G826A, exon6:c.G568A, exon7:c.G949A | 19p13.2 | Likely pathogenic |
| LDLR | rs875989895 | G | A | exonic |  | missense SNV | exon3:c.G311A | 19p13.2 | Likely pathogenic |
| LDLR | rs875989907 | G | A | exonic | NM_000527, NM_001195798, NM_001195799, NM_001195803 | missense SNV | exon4:c.G673A, exon4:c.G415A, exon5:c.G796A | 19p13.2 | Likely pathogenic |
| LDLR | rs748944640 | G | A | exonic |  | missense SNV | exon3:c.G295A, exon4:c.G418A | 19p13.2 | Likely pathogenic |
| MUT | rs879253830 | G | C | exonic | NM_000255 | missense SNV | exon3:c.C560G | 6p12.3 | Pathogenic |
| MYH11 | rs375159635 | C | T | exonic | NM_001040113, NM_001040114, NM_002474, NM_022844 | missense SNV | exon2:c.G301A | 16p13.11 | Likely pathogenic |
| MYH11 | rs751495086 | C | T | exonic |  | missense SNV | exon38:c.G5375A, exon39:c.G5396A | 16p13.11 | Likely pathogenic |
| NDUFS1 | rs767122069 | C | T | exonic | NM_001199981, NM_001199982, NM_001199983, NM_001199984, NM_005006 | missense SNV | exon16:c.G1774A, exon18:c.G1999A, exon18:c.G1936A, exon19:c.G2149A, exon19:c.G2107A | 2q33.3 | Likely pathogenic |
| PAX8 | rs104893657 | C | T | exonic | NM_003466, NM_013952, NM_013953, NM_013992 | missense SNV | exon3:c.G92A | 2q13 | Pathogenic; |
| PIGA | rs587777396 | G | A | exonic | NM_002641 | missense SNV | exon2:c.C355T | Xp22.2 | Pathogenic/ Likely pathogenic |
| POLG | rs770115219 | G | A | exonic | NM_001126131, NM_002693 | missense SNV | exon3:c.C694T | 15q26.1 | Pathogenic |
| PPOX | rs41270025 | G | A | exonic | NM_000309, NM_001122764 | missense SNV | exon6:c.G503A | 1q23.3 | Pathogenic |
| PROK2 | rs121434272 | G | A | exonic | NM_001126128, NM_021935 | missense SNV | exon2:c.C217T | 3p13 | Pathogenic |
| RAD54B | rs114216685 | T | C | exonic | NM_001205263, NM_012415 | missense SNV | exon8:c.A1226G, exon10:c.A1778G | 8q22.1 | Pathogenic; |
| RBM20 | rs267607003 | C | T | exonic | NM_001134363 | missense SNV | exon9:c.C1913T | 10q25.2 | Pathogenic |
| RS1 | rs281865355 | C | T | exonic | NM_000330 | missense SNV | exon6:c.G590A | Xp22.13 | Pathogenic/ Likely pathogenic |
| RYR1 | rs886039586 | A | G | exonic | NM_000540, NM_001042723 | missense SNV | exon99:c.A14401G, exon100:c.A14416G | 19q13.2 | Pathogenic |
| RYR1 | rs138874610 | G | A | exonic |  | missense SNV | exon18:c.G2122A | 19q13.2 |  |
| RYR1 | rs747644094 | C | T | exonic |  | missense SNV | exon36:c.C5926T | 19q13.2 |  |
| SCN2A | rs794727152 | G | A | exonic | NM_001040142, NM_001040143, NM_021007 | missense SNV | exon14:c.G2558A | 2q24.3 | Pathogenic |
| SCN2A | rs1057518350 | G | A | exonic |  | missense SNV | exon9:c.G1351A, exon10:c.G1351A | 2q24.3 | Likely pathogenic |
| SCN4A | rs121908552 | C | T | exonic | NM_000334 | missense SNV | exon9:c.G1333A | 17q23.3 | Pathogenic |
| SCN4A | rs779953580 | C | T | exonic |  | missense SNV | exon22:c.G4010A | 17q23.3 | Likely pathogenic |
| SCN5A | rs150264233 | G | A | exonic | NM_000335, NM_001099404, NM_001099405, NM_001160160, NM_001160161, NM_198056 | missense SNV | exon27:c.C5657T, exon27:c.C5549T, exon28:c.C5708T, exon28:c.C5711T, exon28:c.C5612T, exon28:c.C5711T | 3p22.2 | Likely pathogenic |
| SCN5A | rs766875593 | C | T | exonic |  | missense SNV | exon27:c.G5614A, exon27:c.G5506A, exon28:c.G5665A, exon28:c.G5668A, exon28:c.G5569A | 3p22.2 | Likely pathogenic |
| SCN5A | rs199473297 | G | A | exonic |  | missense SNV | exon27:c.C5072T, exon27:c.C4964T, exon28:c.C5123T, exon28:c.C5126T, exon28:c.C5027T, exon28:c.C5126T | 3p22.2 | Likely pathogenic |
| SDHA | rs200397144 | C | T | exonic | NM_004168 | missense SNV | exon13:c.C1753T | 5p15.33 | Pathogenic |
| SDHA | rs112307877 | CTT | C | exonic |  | frameshift deletion | exon15:c.1944_1945del | 5p15.33 | Likely pathogenic |
| SGCA | rs28933693 | C | T | exonic | NM_000023, NM_001135697 | missense SNV | exon3:c.C229T | 17q21.33 | Pathogenic |
| SPG11 | rs312262720 | CAT | C | exonic | NM_001160227, NM_025137 | frameshift deletion | exon4:c.733_734del | 15q21.1 | Pathogenic |
| SPG7 | rs199804717 | G | A | exonic | NM_003119 | missense SNV | exon13:c.G1771A | 16q24.3 | Likely pathogenic |
| SPG7 | . | C | T | exonic | NM_003119, NM_199367 | missense SNV | exon9:c.C1198T | 16q24.3 | Likely pathogenic |
| TK2 | rs281865496 | C | T | exonic | NM_001172643, NM_001172644, NM_001172645, NM_001271934, NM_001272050, NM_004614 | missense SNV | exon7:c.G500A, exon7:c.G521A, exon8:c.G482A, exon8:c.G575A, exon9:c.G428A | 16q21 | Pathogenic |

| TP53 | rs587782664 | C | T | exonic | NM_000546, NM_001126112, NM_001126113, NM_001126114, NM_001126115, NM_001126116, NM_001126117, NM_001126118, NM_001276695, NM_001276696, NM_001276697, NM_001276698, NM_001276699, NM_001276760, NM_001276761 | missense SNV | exon3:c.G315A, exon3:c.G234A, exon6:c.G594A, exon7:c.G711A, exon7:c.G594A, | 17p13.1 | Likely pathogenic |
| --- | --- | --- | --- | --- | --- | --- | --- | --- | --- |
| TP53 | rs121912657 | C | A | exonic |  | missense SNV | exon4:c.G418T, exon4:c.G337T, exon7:c.G697T, exon8:c.G814T, exon8:c.G697T | 17p13.1 | Pathogenic |
| TP53 | rs1057519991 | T | A | exonic |  | missense SNV | exon1:c.A140T, exon1:c.A59T, exon4:c.A419T, exon5:c.A536T, exon5:c.A419T | 17p13.1 | Likely pathogenic |
| TP53 | rs121912660 | C | G | exonic |  | missense SNV | exon4:c.G443C, exon4:c.G362C, exon7:c.G722C, exon8:c.G839C, exon8:c.G722C | 17p13.1 | Pathogenic |
| TTN | rs372496072 | C | T | exonic | NM_001256850, NM_001267550, NM_003319, NM_133378, NM_133432, NM_133437 | missense SNV | exon150:c.G41255A, exon151:c.G41630A, exon151:c.G41831A, exon271:c.G60746A, exon272:c.G63527A, exon322:c.G68450A | 2q31.2 | Likely pathogenic |
| TTN | rs35683768 | C | A | exonic |  | missense SNV | exon3:c.G178T, | 2q31.2 | Likely pathogenic |
| TTN | rs397517600 | C | T | exonic |  | missense SNV | exon96:c.G23257A, exon97:c.G23632A, exon97:c.G23833A, exon217:c.G42748A, exon218:c.G45529A, exon268:c.G50452A | 2q31.2 | Likely pathogenic |

| TTN | rs397517778 | C | G | exonic |  | missense SNV | exon182:c.G71907C, exon183:c.G72282C, exon183:c.G72483C, exon303:c.G91398C, exon304:c.G94179C, exon354:c.G99102C | 2q31.2 | Likely pathogenic |
| --- | --- | --- | --- | --- | --- | --- | --- | --- | --- |
| TUBB3 | rs886041459 | C | T | exonic | NM_001197181, NM_006086 | missense SNV | exon4:c.C473T, exon4:c.C689T | 16q24.3 | Pathogenic |
| USH2A | rs201527662 | A | C | exonic | NM_007123, NM_206933 | missense SNV | exon13:c.T2802G | 1q41 | Pathogenic; |
| WFS1 | rs774265764 | G | A | exonic | NM_001145853, NM_006005 | missense SNV | exon8:c.G1673A | 4p16.1 | Likely pathogenic |
| ZC4H2 | rs1064795680 | C | T | exonic | NM_001178032, NM_001178033, NM_001243804, NM_018684 | missense SNV | exon2:c.G131A, exon2:c.G200A | Xq11.2 | Likely pathogenic |

**Table F4** GO Annotation for pathogenic and likely pathogenic genes

| **Category** | **description** | **Gene Ratio** | **PValue** | **Genes** | **Count** |
| --- | --- | --- | --- | --- | --- |
| **GOTERM_BP** | muscle contraction | 5/52 | 3.09×10-4 | RYR1, SGCA,  MYH11, SCN4A, TTN | 5 |
| **GOTERM_BP** | sodium ion transmembrane transport | 4/52 | 1.41×10-3 | SCN4A, SCN5A,  SCN2A, ABCB11 | 4 |
| **GOTERM_BP** | regulation of postsynaptic  membrane potential | 3/52 | 2.01×10-3 | SCN4A, SCN5A, SCN2A | 3 |
| **GOTERM_BP** | visual perception | 5/52 | 3.20×10-3 | RS1, GJA3, WFS1,  ABCA4, USH2A | 5 |
| **GOTERM_BP** | membrane depolarization  during action potential | 3/52 | 3.25×10-3 | SCN4A, SCN5A, SCN2A | 3 |
| **GOTERM_BP** | neuronal action potential | 3/52 | 3.25×10-3 | SCN4A, SCN5A, SCN2A | 3 |
| **GOTERM_BP** | skeletal muscle myosin  thick filament assembly | 2/52 | 9.08×10-3 | MYH11, TTN | 2 |
| **GOTERM_BP** | morphogenesis of an epithelial fold | 2/52 | 1.20×10-2 | AR, EGFR | 2 |
| **GOTERM_BP** | hair cell differentiation | 2/52 | 1.80×10-2 | ERCC2, USH2A | 2 |
| **GOTERM_BP** | neurological system process | 2/52 | 2.40×10-2 | CHRNA4, WFS1 | 2 |
| **GOTERM_BP** | cardiac muscle fiber development | 2/52 | 2.40×10-2 | MYH11, TTN | 2 |
| **GOTERM_BP** | cell proliferation | 5/52 | 2.48×10-2 | AR, ERCC2,  PROK2, TP53, EGFR | 5 |
| **GOTERM_BP** | sodium ion transport | 3/52 | 2.51×10-2 | SCN4A, SCN5A, SCN2A | 3 |
| **GOTERM_BP** | ER overload response | 2/52 | 2.99×10-2 | WFS1, TP53 | 2 |
| **GOTERM_BP** | UV protection | 2/52 | 3.59×10-2 | ERCC2, ERCC5 | 2 |
| **GOTERM_BP** | response to oxidative stress | 3/52 | 4.40×10-2 | CHRNA4, ERCC2, EGFR | 3 |
| **GOTERM_BP** | positive regulation of  gene expression | 4/52 | 4.52×10-2 | AR, EDA,  TP53, LDLR | 4 |
| **GOTERM_BP** | nervous system development | 4/52 | 5.64×10-2 | JAG1, ZC4H2,  SPG7, SDHA | 4 |
| **GOTERM_BP** | nucleotide-excision repair,  preincision complex stabilization | 2/52 | 6.19×10-2 | ERCC2, ERCC5 | 2 |
| **GOTERM_BP** | nucleotide-excision repair,  DNA incision, 3'-to lesion | 2/52 | 6.76×10-2 | ERCC2, ERCC5 | 2 |
| **GOTERM_BP** | positive regulation of transcription,  DNA-templated | 5/52 | 7.07×10-2 | AR, PAX8, INSR,  ERCC2, TP53 | 5 |
| **GOTERM_BP** | multicellular organism development | 5/52 | 7.30×10-2 | FLG, RS1, JAG1,  PAX8, TP53 | 5 |
| **GOTERM_BP** | membrane depolarization | 2/52 | 7.32×10-2 | CHRNA4, SCN5A | 2 |
| **GOTERM_BP** | positive regulation of phosphorylation | 2/52 | 7.33×10-2 | AR, EGFR | 2 |
| **GOTERM_BP** | DNA biosynthetic process | 2/52 | 7.60×10-2 | TK2, POLG | 2 |
| **GOTERM_BP** | peptidyl-tyrosine phosphorylation | 3/52 | 7.87×10-2 | INSR, EGFR, TTN | 3 |
| **GOTERM_BP** | neuromuscular junction development | 2/52 | 8.17×10-2 | ZC4H2, COL4A5 | 2 |
| **GOTERM_BP** | nucleotide-excision repair,  preincision complex assembly | 2/52 | 8.45×10-2 | ERCC2, ERCC5 | 2 |
| **GOTERM_BP** | sarcomere organization | 2/52 | 8.45×10-2 | LDB3, TTN | 2 |
| **GOTERM_BP** | transport | 4/52 | 8.88×10-2 | AR, GJA3,  ABCA4, ABCB11 | 4 |
| **GOTERM_BP** | aging | 3/52 | 8.96×10-2 | ERCC2, KRT14, POLG | 3 |
| **GOTERM_BP** | response to gamma radiation | 2/52 | 9.00×10-2 | TP53, POLG | 2 |
| **GOTERM_BP** | response to hypoxia | 3/52 | 9.60×10-2 | RYR1, CHRNA4, ERCC2 | 3 |
| **GOTERM_BP** | photoreceptor cell maintenance | 2/52 | 9.83×10-2 | ABCA4, USH2A | 2 |
| **GOTERM_CC** | voltage-gated sodium channel complex | 3/52 | 6.84×10-4 | SCN4A, SCN5A, SCN2A | 3 |
| **GOTERM_CC** | membrane | 15/52 | 2.25×10-3 | RYR1, EDA, JAG1,  CHRNA4, INSR, ABCA4,  ABCB11, SDHA, EGFR,  PIGA, NDUFS1, GUSB,  LDLR, ABCD1, LBR | 15 |
| **GOTERM_CC** | Z disc | 4/52 | 4.39×10-3 | RYR1, LDB3,  SCN5A, TTN | 4 |
| **GOTERM_CC** | integral component of membrane | 24/52 | 7.78×10-3 | RYR1, FBN2, CASR,  EDA, JAG1, WFS1,  CHRNA4, HOGA1,  SPG7, ABCB11,  USH2A, EGFR, AR,  COQ2, SGCA, GJA3,  PIGA, ERCC5, SCN5A,  SCN2A, LDLR, TP53,  ABCD1, LBR | 24 |
| **GOTERM_CC** | dendrite | 5/52 | 1.42×10-2 | CHRNA4, WFS1,  TUBB3, ACAD9, SPG11 | 5 |
| **GOTERM_CC** | integral component of  plasma membrane | 10/52 | 1.56×10-2 | RYR1, CASR, JAG1,  EDA, INSR, ABCA4,  SCN4A, SCN2A,  ABCB11, LDLR | 10 |
| **GOTERM_CC** | apical part of cell | 3/52 | 1.87×10-2 | EDA, ABCB11, LDLR | 3 |
| **GOTERM_CC** | sarcolemma | 3/52 | 2.36×10-2 | RYR1, SGCA, SCN5A | 3 |
| **GOTERM_CC** | holo TFIIH complex | 2/52 | 3.03×10-2 | ERCC2, ERCC5 | 2 |
| **GOTERM_CC** | mitochondrion | 9/52 | 3.04×10-2 | ACAD9, NDUFS1,  HOGA1, SPG7, SDHA,  MUT, TP53,  ABCD1, POLG | 9 |
| **GOTERM_CC** | muscle myosin complex | 2/52 | 4.39×10-2 | MYH11, TTN | 2 |
| **GOTERM_CC** | receptor complex | 3/52 | 4.92×10-2 | INSR, LDLR, EGFR | 3 |
| **GOTERM_CC** | integral component of mitochondrial  inner membrane | 2/52 | 5.45×10-2 | COQ2, PPOX | 2 |
| **GOTERM_CC** | I band | 2/52 | 6.24×10-2 | RYR1, TTN | 2 |
| **GOTERM_CC** | mitochondrial matrix | 4/52 | 6.34×10-2 | TK2, NDUFS1,  MUT, TP53 | 4 |
| **GOTERM_CC** | intracellular membrane-bounded organelle | 5/52 | 7.03×10-2 | FLG, EDA, INSR,  ABCA4, GUSB | 5 |
| **GOTERM_CC** | endosome membrane | 3/52 | 9.46×10-2 | INSR, LDLR, EGFR | 3 |
| **GOTERM_CC** | transcription factor TFIID complex | 2/52 | 9.60×10-2 | ERCC2, TP53 | 2 |
| **GOTERM_MF** | protease binding | 5/52 | 2.43×10-4 | RYR1, TP53, LDLR,  POLG, TTN | 5 |
| **GOTERM_MF** | calmodulin binding | 6/52 | 2.58×10-4 | RYR1, WFS1,  MYH11, SCN5A,  EGFR, TTN | 6 |
| **GOTERM_MF** | voltage-gated sodium channel activity | 3/52 | 1.64×10-3 | SCN4A, SCN5A, SCN2A | 3 |
| **GOTERM_MF** | enzyme binding | 6/52 | 3.23×10-3 | AR, SCN5A, TP53,  ABCD1, EGFR, TTN | 6 |
| **GOTERM_MF** | ATP binding | 12/52 | 4.38×10-3 | INSR, ERCC2, ABCA4,  MYH11, TK2, SPG7,  ABCB11, TP53, ABCD1,  EGFR, TTN, RAD54B | 12 |
| **GOTERM_MF** | ATPase activity, coupled to transmembrane  movement of substances | 3/52 | 7.80×10-3 | ABCA4, ABCB11, ABCD1 | 3 |
| **GOTERM_MF** | protein binding | 36/52 | 1.19×10-2 | FLG, FBN2, RYR1,  EDA, KMT2C, ZC4H2,  LDB3, SPG7, EGFR,  TTN, RAD54B, SGCA,  TUBB3, MYH11, SCN5A,  SPG11, ABCD1, LBR,  LDLR, POLG, CASR,  EIF2B3, JAG1, WFS1,  INSR, ACAD9, SDHA,  USH2A, AR, PAX8,  PIGA, ERCC2, KRT14,  ERCC5, NDUFS1, TP53 | 36 |
| **GOTERM_MF** | flavin adenine dinucleotide binding | 3/52 | 1.60×10-2 | ACAD9, SDHA, PPOX | 3 |
| **GOTERM_MF** | calcium ion binding | 7/52 | 2.04×10-2 | RYR1, FLG, FBN2,  JAG1, SGCA,  LDLR, TTN | 7 |
| **GOTERM_MF** | double-stranded DNA binding | 3/52 | 2.49×10-2 | ERCC5, TP53, EGFR | 3 |
| **GOTERM_MF** | muscle alpha-actinin binding | 2/52 | 2.69×10-2 | LDB3, TTN | 2 |
| **GOTERM_MF** | receptor signaling protein tyrosine  kinase activity | 2/52 | 2.98×10-2 | INSR, EGFR | 2 |
| **GOTERM_MF** | electron carrier activity | 3/52 | 3.02×10-2 | ACAD9, NDUFS1, SDHA | 3 |
| **GOTERM_MF** | protein N-terminus binding | 3/52 | 3.47×10-2 | ERCC2, ERCC5, TP53 | 3 |
| **GOTERM_MF** | oxidoreductase activity, acting on  the CH-CH group of donors | 2/52 | 5.30×10-2 | ACAD9, LBR | 2 |
| **GOTERM_MF** | ubiquitin protein ligase binding | 4/52 | 5.57×10-2 | WFS1, SCN5A,  TP53, EGFR | 4 |
| **GOTERM_MF** | actin filament binding | 3/52 | 6.03×10-2 | MYH11, EGFR, TTN | 3 |
| **GOTERM_MF** | protein tyrosine kinase activity | 3/52 | 6.11×10-2 | INSR, EGFR, TTN | 3 |
