# Supplementary material for: Application value of whole exome sequencing in screening and identifying novel mutations of hypopharyngeal cancer
Source: Sci Rep. 2023 Jan 3;13:107. doi: 10.1038/s41598-022-27273-w (PMC9810646; doi:10.1038/s41598-022-27273-w)
Supplement: Supplementary file 2 — Supplementary Figures. [file 41598_2022_27273_MOESM2_ESM.pdf]

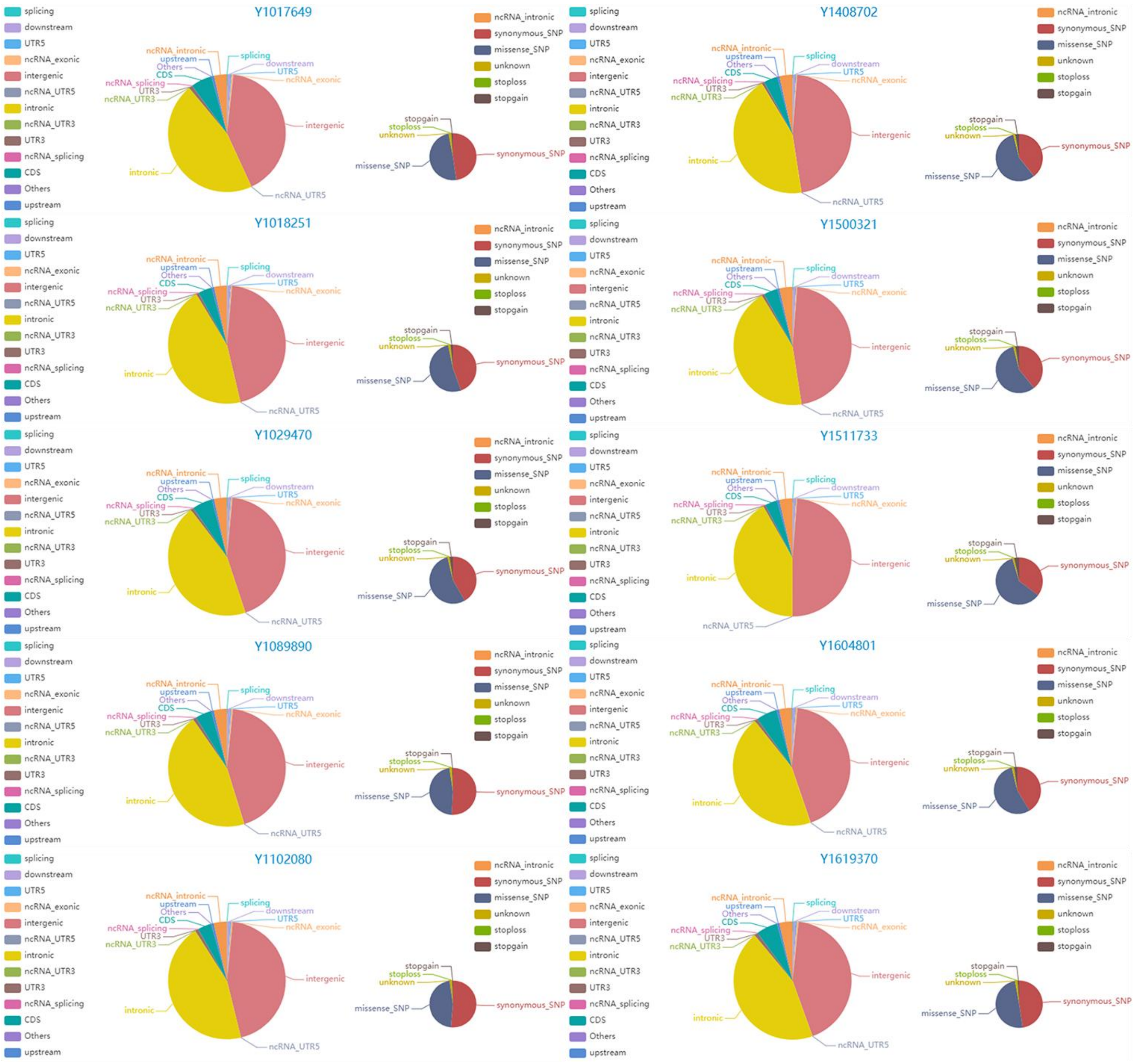

Supplementary figure 1 Single-nucleotide variants (SNVs) in 10 patients with hypopharyngeal carcinoma

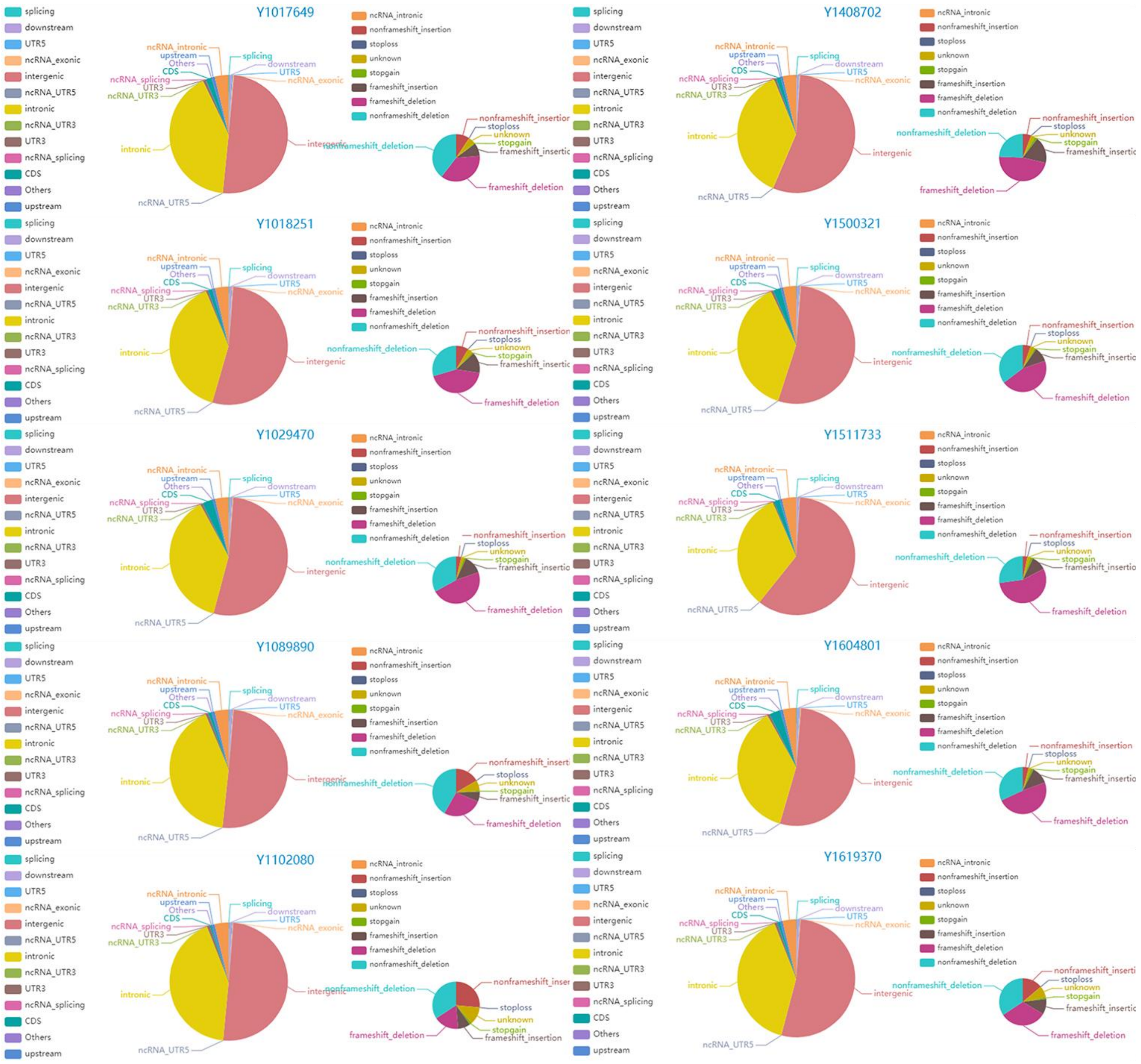

Supplementary figure 2 Insertions and deletions (INDELs) in 10 patients with hypopharyngeal carcinoma
